# Hierarchical probabilistic models for multiple gene/variant associations based on next-generation sequencing data

**DOI:** 10.1093/bioinformatics/btx355

**Published:** 2017-05-31

**Authors:** Dimitrios V Vavoulis, Jenny C Taylor, Anna Schuh

**Affiliations:** 1 The Nuffield Division of Clinical Laboratory Sciences, University of Oxford, Oxford, UK; 2 The Wellcome Trust Centre for Human Genetics, University of Oxford, Oxford, UK; 3 National Health Service Translational Molecular Diagnostics Centre, Oxford University Hospitals, John Radcliffe Hospital, Oxford, UK; 4 National Institute for Health Research Oxford Biomedical Research Centre, Oxford, UK; 5 Department of Oncology, University of Oxford, Oxford, UK

## Abstract

**Motivation:**

The identification of genetic variants influencing gene expression (known as *expression quantitative trait loci* or *eQTLs*) is important in unravelling the genetic basis of complex traits. Detecting multiple eQTLs *simultaneously* in a population based on paired DNA-seq and RNA-seq assays employs two competing types of models: models which rely on appropriate transformations of RNA-seq data (and are powered by a mature mathematical theory), or *count-based models*, which represent digital gene expression explicitly, thus rendering such transformations unnecessary. The latter constitutes an immensely popular methodology, which is however plagued by mathematical intractability.

**Results:**

We develop tractable count-based models, which are amenable to efficient estimation through the introduction of latent variables and the appropriate application of recent statistical theory in a sparse Bayesian modelling framework. Furthermore, we examine several transformation methods for RNA-seq read counts and we introduce *arcsin*, *logit* and *Laplace smoothing* as preprocessing steps for transformation-based models. Using natural and carefully simulated data from the *1000 Genomes* and *gEUVADIS* projects, we benchmark both approaches under a variety of scenarios, including the presence of noise and violation of basic model assumptions. We demonstrate that an arcsin transformation of Laplace-smoothed data is at least as good as state-of-the-art models, particularly at small samples. Furthermore, we show that an over-dispersed Poisson model is comparable to the celebrated Negative Binomial, but much easier to estimate. These results provide strong support for transformation-based versus count-based (particularly Negative-Binomial-based) models for eQTL mapping.

**Availability and implementation:**

All methods are implemented in the free software *eQTLseq*: https://github.com/dvav/eQTLseq

**Supplementary information:**

Supplementary data are available at *Bioinformatics* online.

## 1 Introduction

The identification of genetic variants affecting gene expression (known as *expression quantitative trait loci* or *eQTLs*) is an important step in unravelling the genetic basis of complex traits, including diseases (Albert and Kruglyak, 2015; Cookson *et al.*, 2009; Joehanes *et al.*, 2017). Powered by next-generation sequencing (NGS) and the simultaneous genome-wide profiling of genetic variation and gene expression, this task poses novel statistical challenges due to the idiosyncratic nature of the data generated by these technologies. Count data produced by assays such as RNA-seq (Wang *et al.*, 2009), constitute a digital measure of gene expression, thus making methodologies developed for continuous microarray data not directly applicable.

A straightforward approach to eQTL mapping using RNA-seq would be to transform digital expression data (Zwiener *et al.*, 2014) and then proceed using methodologies developed for micro-arrays, which usually assume normally distributed data (Bottolo *et al.*, 2011; Cheng *et al.*, 2014; Flutre *et al.*, 2013; Shabalin, 2012; Yi and Xu, 2008). The basic obstacle in directly applying such methods on normalized RNA-seq data are the high degree of skewness, extreme values and a non-trivial mean-variance relationship, which commonly characterize such data. A simple, albeit imperfect, approach to ameliorate these effects is the application of a logarithmic transformation, making sure that zero counts are appropriately handled in order to avoid infinities. Two further options are power transformations, such as the *Box-Cox* transformation (Box and Cox, 1964), and rank-based transformations, such as the *Blom* transformation (Beasley *et al.*, 2009), both of which aim to make the data more normal-like. While the aforementioned transformations are not specific to RNA-seq, variance-stabilizing approaches that explicitly model the mean-variance relationship in such data are provided by specialized software, such as *DESeq2* (functions *rlog* and *vst*) (Love *et al.*, 2014) and *limma* (function *voom*) (Law *et al.*, 2014). The practical advantage of using appropriate data transformations is the immediate availability of analytical methods, which (being built around the assumption of normally distributed data) are powered by a tractable mathematical theory.

An alternative approach is to develop statistical methods that specifically address the discrete nature of digital expression data (Kumasaka *et al.*, 2016; Sun and Hu, 2013). Explicitly modeling counts has been quite popular in the study of differential gene expression, and several methods have been developed for this purpose based on Poisson, Binomial and, especially, Negative Binomial distributions (Kvam *et al.*, 2012; Seyednasrollah *et al.*, 2015; Soneson and Delorenzi, 2013). These models directly address non-normality (particularly at small count numbers) and non-linear mean-variance trends, and they often adopt some form of information sharing between genes for shrinking dispersion estimates. A natural strategy to achieve the latter is by imposing a prior distribution on the dispersion parameter within a Bayesian inference framework and ‘let the data decide’ on an appropriate amount of shrinkage (Vavoulis *et al.*, 2015; Wu *et al.*, 2013).

Regardless of which approach is used to model digital expression data, the problem of finding associations with particular genetic variants (i.e. eQTLs) can be expressed as a regression problem, where gene expression is treated as the response variable and the genotypes of the variants as the explanatory variables. The aim of subsequent analysis is to estimate a set of coefficients that capture the strength of all possible associations between variants and genes. As such, the problem of eQTL mapping is intimately related to genetic (possibly genome-wide) association studies and, indeed, searching for eQTLs can be thought of as a genetic association task, where gene expression plays the role of the phenotype. The simplest approach is to examine each gene-variant pair independently by expressing their relationship as a simple, univariate regression problem (Kumasaka *et al.*, 2016; Shabalin, 2012; Sun and Hu, 2013). This is perhaps the most computationally feasible choice, when a large number of gene-variant pairs is examined, but it typically requires some form of multiplicity correction in order to retain an acceptable Type I error rate and it bears the risk of missing possible synergistic effects of multiple variants on gene expression. If such a risk is not acceptable and, especially, if a set of genes and variants has been pre-selected (for example, on the basis of clinical criteria), an alternative would be to model the effect of multiple variants on the expression of multiple genes simultaneously, in which case a multivariate/multiple regression framework becomes appropriate (Bottolo *et al.*, 2011; Cheng *et al.*, 2014; Flutre *et al.*, 2013; Yi and Xu, 2008;).

In this paper, we develop two classes of statistical models for detecting *simultaneously* multiple associations between gene expression and genomic polymorphisms in a population, as measured by paired DNA-seq and RNA-seq assays. The first class involves Poisson, Binomial and Negative Binomial models, which explicitly model digital gene expression as a function of genetic variation. The second class involves a Normal/Gaussian model, which relies on appropriate transformations of gene expression data. All models are embedded in a Bayesian multiple/multivariate regression and variable selection framework, which permits for fair comparison between them. Traditionally, Bayesian inference in regression models involving the Negative Binomial distribution has been discouraged due to the intractable form of the likelihood function (Polson *et al.*, 2013). An important contribution of this paper is expressing the posterior probability of multiple gene-variant associations in the Negative Binomial and the other count-based models in a convenient mathematical form through the introduction of latent variables, which facilitates sparse Bayesian learning. A second important contribution, is the introduction of Laplace smoothing (Chen and Goodman, 1999) and the *arcsin* transformation (Warton and Hui, 2011) for pre-processing digital gene expression data. Using carefully simulated RNA-seq and genotype data and natural data from the *1000 Genomes* (1000 Genomes Project Consortium *et al.*, 2015) and *gEUVADIS* projects (Lappalainen *et al.*, 2013), we show that this pre-processing step in combination with a Normal model demonstrates top performance in a variety of scenarios, particularly at small samples sizes. The over-dispersed Poisson and Negative Binomial models also show excellent performance, when the sample size is sufficiently large. We examine model behavior in a variety of scenarios, including the presence of two different types of noise, violation of model assumptions, various association strengths and patterns of eQTL distribution. All methods are implemented in the freely available Python software *eQTLseq* (https://github.com/dvav/eQTLseq).

## 2 Materials and methods

### 2.1 Model overview

**Fig. 1.**
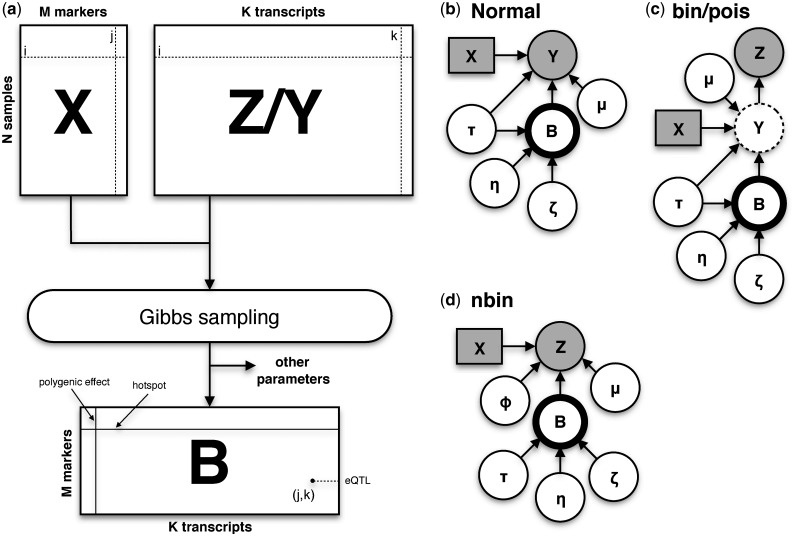
Workflow and statistical models. (**a**) A matrix of genotypes **X** and a matrix of untransformed (**Z**) or transformed (**Y**) expression data are used in the estimation of a matrix of coefficients **B**, which captures the associations between *M* variants and *K* transcripts in a population of *N* samples. (**b**, **c**, **d**) Dependencies between random variables in Normal (b), over-dispersed Poisson (c), over-dispersed Binomial (c) and Negative Binomial (d) models. ***shaded rectangle and circles*:** observed data (genotypes and gene expression); ***dash circle:*** latent (unobserved) data; **thick circle:** the matrix of association coefficients **B**

Below, we briefly describe the main elements of the proposed models. A detailed mathematical treatise and associated inference methods is given in the Supplementary Material. *eQTLseq* (Fig. 1a) implements a number of hierarchical probabilistic models, which represent gene expression as a function of genetic variation [Fig. 1(b–d)]. Three of these models (Binomial or *bin*, Negative Binomial or *nbin* and Poisson or *pois*) take explicitly into account the discrete nature of expression data measured in, for example, RNA-seq assays, while a fourth model is based on the Normal distribution and it relies on appropriate transformations of such expression data (see *Data transformations*). We assume that genotype data and count data measuring transcript abundance for *N* samples is summarized in an *N *×* M* matrix **X** and an *N *×* K* matrix **Z**, respectively, where *M* is the number of genetic markers and *K* is the number of transcripts. Under an additive genetic model, each element of **X** takes values in the set {0, 1, 2} indicating the number of minor alleles at a specific locus. *eQTLseq* takes matrices **X** and **Z** (or **Y**, a transformed version of **Z**) as input and employs *Gibbs sampling* (Andrieu *et al.*, 2003) to estimate an *M *×* K* matrix of regression coefficients **B**. The elements *β_jk_* of this matrix can be positive, negative or zero indicating positive, negative or no effect of marker *j* on the expression of transcript *k*. We assume that **B** is sparse (i.e. most elements *β_jk_* are zero), which implies that most markers in **X** have no influence on transcript abundance. In all models, sparsity is induced by assuming the following prior for each *β_jk_*:

$$p\left(\beta_{jk}\right)∼N\left(0,\tau_{k}^{-1}\zeta_{jk}^{-1}\eta_{j}^{-1}\right)$$

where $N\left(0,\xi^{-1}\right)$ is the Normal distribution with mean 0 and variance $\xi^{-1}$, and *τ_k_*, *ζ_jk_* and *η_j_* are precision parameters each following Jeffrey’s prior. Jeffrey’s prior was chosen because it is non-informative, it is invariant under re-parametrization, it has strong sparsity-inducing properties and, importantly, it is parameter-free, thus yielding excellent performance without the need for parameter adjustments (Figueiredo *et al.*, 2002). Small values of the marker-specific parameter *η_j_* imply that marker *j* is likely to influence a large number of genes (indicating the presence of a *hotspot*). Similarly, small values of the transcript-specific parameter *τ_k_* imply a transcript that is likely influenced by many markers (indicating a *polygenic effect* on transcript *k*). The synergistic effect of *τ_k_* and *η_j_* is further refined in a marker- and transcript-specific manner through parameter *ζ_jk_*.

We are particularly interested in the posterior distribution $p\left(\beta_{k}|-\right)$ of the vector of regression coefficients $\beta_{k}$, which captures the effects of *M* genetic markers on the abundance of transcript *k* (notice that the symbol – is a shorthand for all random variables the posterior density of $\beta_{k}$ is conditioned on). A major contribution of this work is that we apply recent statistical theory (Polson *et al.*, 2013) to express this posterior in closed form, which greatly facilitates Bayesian learning. Although this is trivial for the Normal model, it is not obvious for the Binomial and Poisson models and particularly difficult for the Negative Binomial, thus discouraging the use of the latter in a Bayesian multivariate regression setting. Here, we show that for all models, the above posterior is a multivariate Normal distribution, with mean vector $m_{k}$ and precision matrix $A_{k}$. In the case of the Normal model, these are simply functions of the genotypes **X**, the transformed expression data $y_{k}$ for transcript *k* and the precision parameters *τ_k_*, $\zeta_{k}$ and η. In the case of the Binomial and Poisson models, $y_{k}$ is an *N*-vector of normally distributed pseudo-data (i.e. latent or unobserved variables). These pseudo-data decouple the coefficients $\beta_{k}$ from the actually observed non-Normal gene expression data, thus making the above inference possible. Importantly, in the case of the Negative Binomial model, we show that parameters $m_{k}$ and $A_{k}$ are functions of the genotypes **X**, the expression data $z_{k}$ for transcript *k*, the precision parameters *τ_k_*, $\zeta_{k}$ and η and a vector $\omega_{k}$ of auxiliary parameters, which follow a Polya-Gamma distribution.

### 2.2 Data transformations

*eQTLseq* requires appropriately transformed expression data as input, when a Normal model is selected. In this paper, we examine various data transformation methods, including a simple logarithmic transformation (*log*), a Box-Cox transformation (Box and Cox, 1964) (*boxcox*), a Blom transformation (Beasley *et al.*, 2009) (*blom*), the *voom* transformation (Law *et al.*, 2014) provided by the R package *limma* and a variance-stabilizing transformation (*vst*) provided by the R package *DESeq2* (Love *et al.*, 2014). A regularized log transformation provided also by *DESeq2* was excluded, because it becomes prohibitively expensive when datasets with hundreds of samples are used, as in the analyses presented in this paper. For the *log* and *Box-Cox* transformations, a single pseudo-count was added to the data in order to avoid infinities.

Furthermore, we introduce *Laplace* (also known as *Lidstone* or *additive*) *smoothing* (Chen and Goodman, 1999) followed by *arcsin* or *logit* transformations (Warton and Hui, 2011) as a preprocessing step. Additive smoothing is commonly used by naive Bayes classifiers and in natural language processing for smoothing categorical data. The inspiration for applying this type of smoothing stems from viewing an RNA-seq sample as the outcome of a multinomial experiment, where a large number of reads (i.e. the raw size of the library) is ‘assigned’ (i.e. mapped) to a number of ‘categories’ (i.e. transcripts). The smoothed mapping probability of each transcript can be expressed as follows:

$$p_{ij}=\frac{z_{ik}+c}{\sum_{k}z_{ik}+cK}$$

where *z_ik_* is the number of reads for transcript *k* in sample *i*, $\sum_{k}z_{ik}$ is the total number of reads in sample *i* and *c* is a positive number, which equals 1 in this paper. *z_ik_* may be normalized prior to Laplace smoothing, if necessary. Notice that, similarly to *voom*, *p_ik_* lies in the interval (0, 1), but unlike *voom*, $\sum_{k}\pi_{ik}=1$. Also, the above probability lies between the empirical probability $z_{ik}/\sum_{k}z_{ik}$ and the uniform probability 1/K. The smaller the library size and the larger the number of genes, the closer the factor *cK* in the denominator pulls *p_ij_* towards 1/K. The probabilities *p_ij_* are further processed using the *arcsin* or *logit* transformations, which are natural choices for proportions (Warton and Hui, 2011).

## 3 Results

### 3.1 Benchmarks based on simulated data

Benchmarks based on simulated data are essential, because they allow control of the exact conditions under which data is generated. At the same time, it is important that simulated data imitate faithfully the statistical characteristics of natural data. In this paper, we simulate read counts and genotypes using data in the public domain as templates (details are provided in the Supplementary Material). Briefly, artificial pairs of read counts and genotype matrices were generated according to the following protocol: (a) decide the number of samples *N* in the artificial dataset (assuming *K* = 1000 genes and *M* = 100 genetic markers, throughout), (b) decide the strength, directionality and pattern of associations between genes and genetic variants and generate an *M *×* K* matrix of coefficients **B** using an Exponential distribution, (c) generate an *N *×* M* matrix of genotypes **X** from a Binomial distribution using data from the *1000 Genomes* project (1000 Genomes Project Consortium *et al.*, 2015) as template, (d) given **X**, **B** and RNA-seq data from 60 *HapMap* individuals of European descent (Frazee *et al.*, 2011; Montgomery *et al.*, 2010) as template, generate an *N *×* K* matrix of read counts **Z** from a Negative Binomial distribution. Different triplets of **X**, **Z** and **B** matrices were generated reflecting differences in: (i) the strength of gene/variant associations (i.e. effect sizes), (ii) the pattern of associations (e.g. the number of hotspots and polygenic effects), (iii) the type and level of random noise in the expression and genotype data, (iv) the presence or absence of over-dispersion and (v) the sample size. For each combination of the above factors, we generate three random replicates resulting in 1512 artificial datasets (i.e. pairs of **X** and **Z** matrices), which each model (*nbin*, *bin*, *pois*, *log*, *arcsin*, *logit*, *blom*, *boxcox*, *voom*, and *vst*) is assessed on, in a total of 15 120 simulations ran on the *Wellcome Trust Center for Human Genetics* local cluster.

**Fig. 2.**
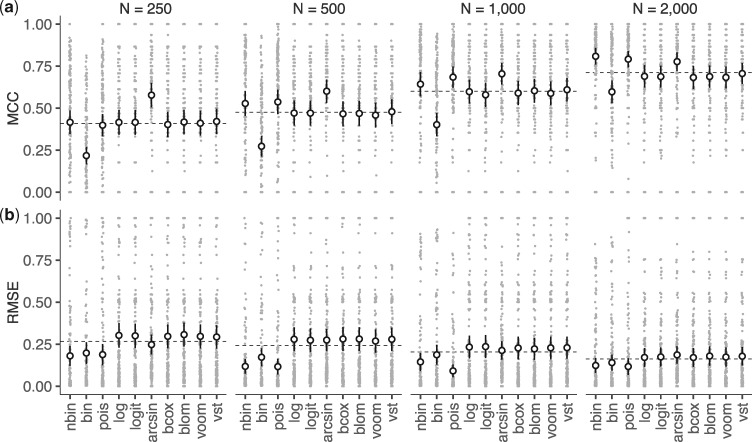
Model performance on simulated data. (**a**) Model comparison using *Matthews correlation coefficient* (MCC) as performance metric. (**b**) Model comparison using the *root mean square error* (RMSE) among true positives as performance metric. For each model at each sample size, we performed 252 simulations (**grey dots**). The **black dots** and **whiskers** indicate the mean and three standard errors on either side of the mean over these simulations

The overall performance of each model at different sample sizes (while the remaining simulation parameters — noise, association strengths, etc. — were left to vary freely, as opposed to being fixed at specific values) is illustrated in Figure 2. We use simulated data with sample sizes equal to 250, 500, 1000 and 2000 subjects and we examine the ability of each model (a) to distinguish correctly between true and false positives and negatives and (b) to estimate correctly the strength of gene/variant associations in the simulated data. For the former, we use *Matthews correlation coefficient* (Matthews, 1975) or *MCC*, which is generally regarded as a balanced measure of performance for binary classifiers, even in cases where the two different classes in the data have very different sizes. For assessing how well each model estimates effect sizes, we calculate the *root mean square error* (RMSE) between estimated and true effect sizes (after these have been appropriately normalized; see Supplementary Material) among the true positives for each model.

As expected, the performance of all models increases with increasing sample size (Fig. 2a). At small sample sizes (*N* = 250), the *arcsin* model clearly has the highest MCC value, while all other models perform similarly. The exception is the Binomial model (*bin*), which demonstrates the smallest MCC value at all sample sizes. As the sample size increases, the performance of the Negative Binomial (*nbin*) and Poisson (*pois*) models increases, slightly overtaking *arcsin* at very large sample sizes (*N = 2000*). All other Normal models perform similarly. At the same time, all count-based models (*bin*, *nbin* and *pois*) are clearly more effective at estimating correctly the strength of gene/variant associations, albeit their distance from Normal models decreases with increasing sample size (Fig. 2b).

The observation that *nbin* and *pois* outperform *arcsin* only at large samples (*N* = 2000) is at least partially explained by the relatively low *true positive rate* (TPR) and *positive predictive value* (PPV) of these two models (see Supplementary Fig. S1) and it should be viewed in terms of model complexity: to fully define the count-based models, we must estimate *N *×* K* more variables than the Normal, which presumably requires larger samples. Interestingly, *pois* performs similarly to or better than *nbin*, although the former is significantly easier and faster to estimate. This is not surprising given that *nbin* can be thought of as a Poisson-Gamma mixture: read counts follow a Poisson distribution with a gamma-distributed rate. *pois* in this study is actually a Poisson-LogNormal mixture: the Poisson rate is log-normally distributed, instead of Gamma. The similar performance of *nbin* and *pois* implies that the latter provides a good approximation to the former. Further results are provided in Supplementary Figures S1–S6, S10 and S11.

### 3.2 Benchmarks based on gEUVADIS data

Next, we tested all models on data from the gEUVADIS project (Lappalainen *et al.*, 2013). This includes mRNA and small RNA sequencing data on 465 lymphoblastoid cell line samples from the *1000 Genomes* project, along with paired data on genomic variation. We kept only variants on bi-allelic loci with MAF larger than 5%, which overlapped any of 1672 human transcription factors available from the FANTOM5 project website (Lizio *et al.*, 2015). These were subsequently annotated with the Variant Effect Predictor (McLaren *et al.*, 2016) (VEP; distributed with the Ensembl Tools v84) and the genotypes of all variants predicted to have HIGH impact were retained for further analysis. gEUVADIS expression data were filtered by removing all transcripts that had on average less than 10 reads across all samples, resulting in two count data matrices with 408 miRNAs and 19 004 mRNAs, respectively.

Ideally, all models should be benchmarked against an objective truth, i.e. the true gene/variant associations in the data. However, as it is usually the case with natural datasets, this truth is not known. Under these circumstances, it is an established practice to adopt a cross-validation approach, which aims to evaluate the predictive value of a particular statistical model or models on independent datasets, i.e. datasets not previously utilized in estimating model parameters or structure. Here, we employ a Monte Carlo cross-validation methodology to objectively compare different models. Briefly, this consists of splitting the paired genotype and gene expression data randomly in two sets, training and validation, with ratio 3:1. Each model is estimated on the training set and its predictive performance is calculated on the validation set. This process is repeated 10 times and an average predictive performance is calculated for each model. As a measure of predictive performance we use the *concordance correlation coefficient* (CCC) (Lin, 1989), which measures the agreement between two sets of values in a scale-independent manner and, thus, ensures fair comparison between different models.

**Fig. 3.**
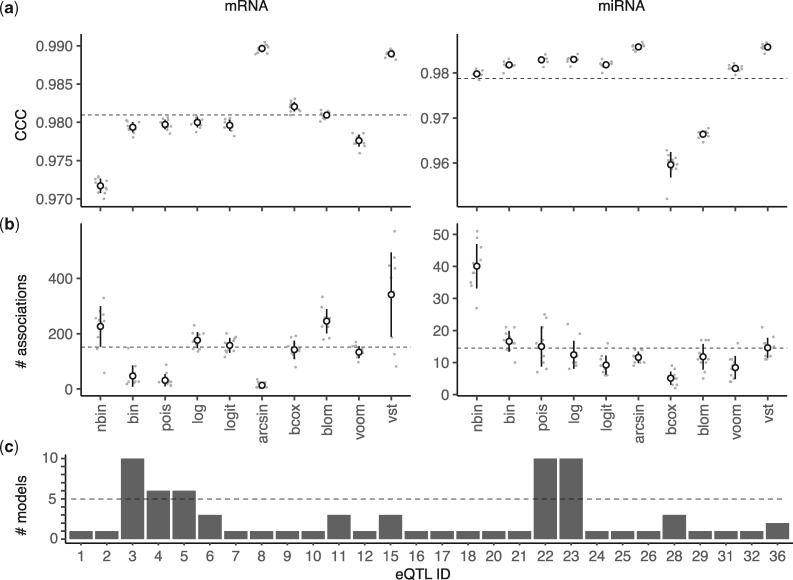
Model performance on *gEUVADIS* data. (**a**) Model comparison on mRNA and miRNA datasets using the *concordance correlation coefficient* (CCC) as performance metric. (**b**) Model comparison with respect to the number of gene/variant associations they identify. In (a) and (b), a Monte Carlo cross-validation protocol with 10 repetitions (**grey points**) was followed for each model and each group (mRNAs or miRNAs). (**c**) Candidate eQTLs and number of supporting models based on all gEUVADIS samples (also, see Table 1). There are *5* eQTLs identified by more than half of the models (**dashed line**)

All models demonstrate very high CCC values (> 0.95) in both mRNAs and miRNAs (Fig. 3a). *arcsin* and *vst* show top performance, with *arcsin* performing slightly better than *vst* for mRNAs. In the same group, *blom* and *boxcox* have average or above average performance, while *bin*, pois, *log* and *logit* perform similarly, just below average. The worst performance is demonstrated by *voom* and, particularly, by the Negative Binomial model. For miRNAs, the situation is the opposite for *blom* and *boxcox*, which now perform worse than all other models. The Binomial, Negative Binomial, Poisson, *log*, *logit*, and *voom* models all perform above average. It is interesting to observe that, in the case of mRNAs, *arcsin* gives the most sparse solutions (i.e. the smallest number of gene/variant associations) among all models, while *vst* gives the least sparse ones followed by *voom* and the Negative Binomial model (Fig. 3b). The Binomial and Poisson models are also quite conservative regarding the number of gene/variant associations they identify. In the case of miRNAs, *boxcox* gives on average the most sparse solutions, followed by *voom* and *logit*, while the Negative Binomial model is clearly the least conservative. We conclude that all models demonstrate excellent predictive performance, with *arcsin* and *vst* being the best among them. At the same time, the *arcsin*, *boxcox*, *Poisson* and *Binomial* models are the most conservative, depending on whether they are applied on mRNA or miRNA datasets. Analysis of all gEUVADIS samples, revealed 28 variants, which have been identified as eQTLs by at least one model (Fig. 3c). Among them, 5 have been consistently selected by more than half of the models (ID: 3, 4, 5, 22, 23; also, see Table 1).

**Table 1.** Variants identified as eQTLs in the gEUVADIS data

ID	dbSNP	CHROM	POS	REF	ALT	MAF	Consequence
3	rs4639011	3	32 030 998	C	T	0.081	stop gained
4	rs6535531	4	76 474 866	T	C	0.496	stop lost
5	rs3217313	5	121 488 635	AT	A	0.186	frameshift
22	rs35575803	19	20 807 177	G	GA	0.730	frameshift
23	rs35999740	19	22 116 015	A	T	0.135	stop gained

## 4 Discussion

Identifying gene/variant associations in a population, based on paired RNA-seq and DNA-seq assays, can be formulated in terms of multiple/multivariate regression, where digital gene expression and genotype data play the role of response and explanatory variables, respectively. We compare two competing approaches for modeling digital gene expression in this context: the first approach employs count-based (Binomial, Negative Binomial and Poisson) models; the second applies a Normal model on appropriately transformed data. Both approaches are embedded in a sparse Bayesian learning framework for regression and variable selection through shrinkage, which simplifies their implementation in software and permits fair comparison between different models. The methodological novelty of our approach is expressing the posterior probability density of gene/variant associations in the form of a multivariate Normal distribution in all models. This is achieved in the case of count-based models through the introduction of latent variables, thus lifting one of the basic reasons discouraging the use of such models (particularly the Negative Binomial one) in a Bayesian learning framework, i.e. mathematical intractability.

Using artificial data modeled closely after data from the *1000 Genomes* project, we demonstrate that a Normal model applied on Laplace-smoothed and *arcsin*-transformed data shows excellent performance particularly at small samples. Negative Binomial and Poisson models are also top performers, with the latter being at least as good as the former, but with smaller computational cost and despite the fact that simulated data assume a Negative Binomial distribution. Further benchmarks on natural data from the gEUVADIS project confirm the top performance of *arcsin*, but also show that the more computationally expensive *vst* is equally good. More generally, the predictive performance of all models is quite high, although not all are equally parsimonious.

Based on these results, we conclude that when mapping eQTLs in a population using NGS data, a Negative Binomial model is not the only or even the best option. This is important because the assumption of a Negative Binomial distribution has been extremely popular in modeling RNA-seq data, particularly in the context of differential gene expression. Instead, researchers can use a Poisson-LogNormal mixture, or a Normal model after appropriately transforming the expression data. The type of transformation that should be used is case-dependent, but our simulations indicate that transforming the RNA-seq data to multinomial probabilities and applying an *arcsin* or even a *logit* (as in the gEUVADIS data) transformation is a good start. In this respect, our conclusions are in agreement with previous work, which highlights the value of Normal models in modeling RNA-seq data (Law *et al.*, 2014; Soneson and Delorenzi, 2013).

The sparsity-inducing priors we use for the regression coefficients **B** are related to the *automatic relevance determination* concept (Tipping, 2001), but other strategies are also possible, including the *Bayesian lasso* (Park and Casella, 2008), the *spike-and-slab* prior (Ishwaran and Rao, 2005) and others (O’Hara and Sillanpaa, 2009). The advantage of our choice of sparse priors is achieving excellent sparsity performance without the need to adjust any parameters. Applying the above statistical framework to possibly thousands of genomic variants involves splitting these variants in groups of several hundreds each, which can be viewed as a compromise between testing each variant independently or all of them simultaneously as a single group (see Supplementary Material for computational considerations). Alternatively, we can employ a dimensionality reduction method, e.g. in the form of *sparse latent factor models* (Knowles and Ghahramani, 2011; West *et al.*, 2003), thus increasing the scale of model applicability, but at the same time introducing the issue of biological interpretability of the latent factors. Further work would also involve modifying the above models to account for strand- and isoform-specific expression data made uniquely available through RNA-seq. Previous work (Kumasaka *et al.*, 2016; Sun and Hu, 2013), which however does not take into account the joint distribution of multiple transcripts and variants, as we do here, should be the logical starting point.

## Supplementary Material

Supplementary Data
