# Supplementary material for: Hierarchical probabilistic models for multiple gene/variant associations based on next-generation sequencing data
Source: Bioinformatics. 2017 May 31;33(19):3058–64. doi: 10.1093/bioinformatics/btx355 (PMC5637939; doi:10.1093/bioinformatics/btx355)
Supplement: Supplementary Data [file suppl_btx355.pdf]

Supplementary material

Hierarchical probabilistic models for multiple gene/variant associations based on next-generation sequencing data

Dimitrios V. Vavoulis[1,2,3,4], Jenny C. Taylor[2,4], and Anna Schuh[3,4,5]

[1]The Nuffield Division of Clinical Laboratory Sciences, University of Oxford, Oxford, UK
[2]The Wellcome Trust Centre for Human Genetics, University of Oxford, Oxford, UK
[3]NHS Translational Molecular Diagnostics Centre, Oxford University Hospitals, John Radcliffe Hospital, Oxford, UK
[4]NIHR Oxford Biomedical Research Centre, Oxford, UK
[5]Department of Oncology, University of Oxford, Oxford, UK

April 29, 2017

1 Supplementary Methods

1.1 Detailed model description

In all cases, we assume an $N \times M$ matrix of genotypes $\mathbf{X}$ and an $N \times K$ matrix of count-data $\mathbf{Z}$, where N, M and K is the number of samples, genetic markers and transcripts, respectively. We also assume an $N \times K$ matrix of expression data $\mathbf{Y}$, which results from transforming $\mathbf{Z}$. We start by describing the Normal model, which Poisson and Binomial models are based on.

1.1.1 Normal model

Assuming $\mathrm{N}(a, b)$ is a univariate Normal distribution with mean a and variance b, this model takes the following form:

$$
\begin{aligned}
y_{ik} &\sim \mathrm{N}\left(\mu_k + \boldsymbol{x}_i^T \boldsymbol{\beta}_k, \tau_k^{-1}\right) \\
\beta_{jk} &\sim \mathrm{N}\left(0, \tau_k^{-1} \zeta_{jk}^{-1} \eta_j^{-1}\right) \\
\mu_k &\sim 1 \\
\tau_k &\sim \tau_k^{-1} \\
\zeta_{jk} &\sim \zeta_{jk}^{-1} \\
\eta_j &\sim \eta_j^{-1}
\end{aligned}
$$

where y_{ik} is the expression level of transcript k for subject i; μ_k is the mean expression level of transcript k; β_{jk} quantifies the effect of marker j on the expression level of transcript k; finally, τ_k, ζ_{jk} and η_j are precision parameters. The dot product $\mathbf{x}_i^T \boldsymbol{\beta}_k$ measures the influence (positive, negative or zero) of the genetic profile of subject i on the mean expression of transcript k.

1.1.2 Poisson model

Assuming $\mathrm{P}(a)$ is a Poisson distribution with mean a, this model takes the following form:

$$
\begin{aligned}
z_{ik} &\sim \mathrm{P}(m_{ik}) \\
\log m_{ik} &= y_{ik}
\end{aligned}
$$

where z_{ik} is the (untransformed) number of counts for transcript k in subject i, m_{ik} is the mean expression level of transcript k for subject i and y_{ik} is a normally distributed latent variable (or pseudo-datum) obeying the Normal model described above. Thus, the Poisson (and Binomial; see below) model can be thought of as an extension of the Normal model through the addition of a Poisson likelihood. Conditional on pseudo-data $\mathbf{Y}$, the observed counts $\mathbf{Z}$ are independent of $\mathbf{B}$, thus greatly simplifying the estimation of the latter, as shown in a subsequent section.

1.1.3 Binomial model

Assuming $\mathrm{B}(a,b)$ is the Binomial distribution with a trials and b probability of success at each trial, the Binomial model is constructed on top of the Normal, similarly to the Poisson:

$$\begin{aligned} z_{ik} &\sim \mathrm{B}(n_i, p_{ik}) \\ \log \frac{p_{ik}}{1-p_{ik}} &= y_{ik} \end{aligned}$$

where $n_i = \sum_k z_{ik}$ is the library size in subject i and p_{ik} is the read mapping probability for transcript k in subject i. As for the Poisson model, y_{ik} is a latent variable following the Normal model.

1.1.4 Negative Binomial model

The final and most elaborate model we examine is the Negative Binomial. Assuming $\mathrm{NB}(a,b)$ is the Negative Binomial distribution with mean a and dispersion parameter b, the model takes the following form:

$$\begin{aligned} z_{ik} &\sim \mathrm{NB}(m_{ik}, \phi_k) \\ \log \phi_k &\sim \mathrm{N}\left(\mu_\phi, \tau_\phi^{-1}\right) \\ \log m_{ik} &= \log \mu_k + \boldsymbol{x}_i^T \boldsymbol{\beta}_k \\ \frac{\mu_k}{\phi_k^{-1} + \mu_k} &\sim \mathrm{Be}\left(\frac{1}{2}, \frac{1}{2}\right) \\ \mu_\phi &\sim 1 \\ \tau_\phi &\sim \tau_\phi^{-1} \end{aligned}$$

where ϕ_k and μ_k are the dispersion and mean expression level of transcript k, respectively. Shrinkage of ϕ_k is achieved by imposing a log-normal prior with mean μ_ϕ and precision τ_ϕ. These parameters in turn have their own priors (uniform and Jeffrey's, respectively) completing the hierarchical model. Importantly, the prior of μ_k is not defined directly; rather, we impose an uninformative Beta prior on the variable $\mu_k / \left(\phi_k^{-1} + \mu_k\right)$, which as we shall see below, simplifies the estimation of μ_k. Notice that β_{jk} (the elements of vector $\boldsymbol{\beta}_k$) are modelled as in the Normal model.

1.2 Inference

Inference in all models is achieved via Gibbs sampling, which requires sampling in turn and repeatedly from the posterior distribution of each random variable in the model, after conditioning on the data and the remaining variables. Below, we describe these conditional posteriors for each model.

1.2.1 Normal model

A tractable mathematical theory exists for the Normal model, which is the simplest to estimate. The necessary posteriors are as follows:

$$\begin{aligned} \mathrm{p}(\boldsymbol{\beta}_k|-) &= \mathrm{N_M}\left(\mathbf{A}_k^{-1}\boldsymbol{X}^T(\boldsymbol{y}_k - \mu_k), \tau_k^{-1}\mathbf{A}_k^{-1}\right) \\ \mathrm{p}(\mu_k|-) &= \mathrm{N}\left(\frac{\sum_i (y_i - \mathbf{x}_i^T \boldsymbol{\beta}_k)}{N}, \tau_k^{-1} N^{-1}\right) \\ \mathrm{p}(\tau_k|-) &= \mathrm{Ga}\left(\frac{M+N}{2}, \frac{\sum_i \left(y_{ik} - \mu_k - \boldsymbol{x}_i^T\boldsymbol{\beta}_k\right)^2}{2} + \frac{\sum_j \beta_{kj}^2 \zeta_{jk}\eta_j}{2}\right) \\ \mathrm{p}(\zeta_{jk}|-) &= \mathrm{Ga}\left(\frac{1}{2}, \frac{\beta_{jk}^2 \tau_k \eta_j}{2}\right) \\ \mathrm{p}(\eta_j|-) &= \mathrm{Ga}\left(\frac{K}{2}, \frac{\sum_k \beta_{jk}^2 \tau_k \zeta_{jk}}{2}\right) \end{aligned}$$

where $\mathrm{Ga}(a,b)$ is a Gamma distribution with shape a and rate b and $\mathrm{N_M}(a,b)$ is a multivariate Normal distribution of dimension M with mean vector a and covariance matrix b. For the transcript-specific precision matrix $\mathbf{A}_k$, we have $\mathbf{A}_k = \boldsymbol{X}^T\boldsymbol{X} + \mathrm{diag}(\eta_1\zeta_{1k}, \ldots, \eta_j\zeta_{jk}, \ldots, \eta_M\zeta_{Mk})$, where $\mathrm{diag}(a)$ indicates an $M \times M$ diagonal matrix with a as its main diagonal.

1.2.2 Poisson model

For the Poisson model, the conditional posterior for each latent variable $\mathrm{p}(y_{ik}|-)$ does not exist in the form of a well-known, easy-to-sample-from distribution. Thus, we employ a Metropolis step at each iteration of the Gibbs sampler, to approximate a sample from this distribution. We use proposals sampled from the prior of y_{ik} (which is Normal; see *Detailed model description*) given the current values of the mean and precision parameters. If y_{ik}^* is such a proposal, it is accepted with probability P_{accept} as follows:

$$P_{accept} = \min\left(1, e^{-(m_{ik}^* - m_{ik})}\left(\frac{m_{ik}^*}{m_{ik}}\right)^{z_{ik}}\right)$$

where $m_{ik} = e^{y_{ik}}$ and $m_{ik}^* = e^{y_{ik}^*}$. Conditional on y_{ik}, the observed counts z_{ik} are independent of the other parts of the model and the remaining posteriors are the same as for the Normal model.

1.2.3 Binomial model

Similarly, for the Binomial model, the conditional posterior $\mathrm{p}(y_{ik}|-)$ is not available in a convenient form and a Metropolis step is employed using proposals y_{ik}^* from the prior, as in the case of the Poisson model. The acceptance probability is:

$$P_{accept} = \min\left(1, \left(\frac{p_{ik}^*}{p_{ik}}\right)^{z_{ik}}\left(\frac{1-p_{ik}^*}{1-p_{ik}}\right)^{n_i - z_{ik}}\right)$$

where $p_{ik}^* = \left(1 + e^{-y_{ik}^*}\right)^{-1}$, $p_{ik} = \left(1 + e^{-y_{ik}}\right)^{-1}$ and n_i is the size of the library for the ith sample (see *Detailed model description* above). As in the Poisson model, given y_{ik} the remaining posteriors are the same as for the Normal.

1.2.4 Negative Binomial model

This model is the most difficult to estimate. The most important methodological contribution of this paper is applying recent statistical theory (Polson *et al.*, 2013) to show that the posterior $\mathrm{p}(\boldsymbol{\beta}_k|-)$ is a multivariate Normal distribution, as shown below:

$$\begin{aligned}
\mathrm{p}(\boldsymbol{\beta}_k|-) &= \mathrm{N_M}\left(\mathbf{A}_k^{-1}\mathbf{X}^T\mathbf{y}_k, \mathbf{A}_k^{-1}\right) \\
\mathrm{p}(\boldsymbol{\omega}_k|-) &= \mathrm{PG}\left(\mathbf{z}_k + \phi_k^{-1}, \log\mu_k + \log\phi_k + \mathbf{X}\boldsymbol{\beta}_k\right) \\
\mathbf{A}_k &= \mathbf{X}^T\boldsymbol{\Omega}_k\mathbf{X} + \mathrm{diag}\left(\tau_k\zeta_{1k}\eta_1, \ldots, \tau_k\zeta_{jk}\eta_j, \ldots, \tau_k\zeta_{Mk}\eta_M\right) \\
\mathbf{y}_k &= \frac{\mathbf{z}_k - \phi_k^{-1} - 2\boldsymbol{\omega}_k\left(\log\mu_k + \log\phi_k\right)}{2} \\
\boldsymbol{\Omega}_k &= \mathrm{diag}\left(\omega_{1k}, \ldots, \omega_{ik}, \ldots, \omega_{Nk}\right)
\end{aligned}$$

where $\boldsymbol{\omega}_k$ is a vector of auxiliary variables sampled from a Polya-Gamma distribution $\mathrm{PG}(b, c)$ with parameters $b > 0$ and $c \in \mathbb{R}$. Briefly, sampling from $\mathrm{p}(\boldsymbol{\beta}_k|-)$ requires first sampling $\boldsymbol{\omega}_k$ from a Polya-Gamma distribution given the current value of $\boldsymbol{\beta}_k$, followed by sampling a new value of $\boldsymbol{\beta}_k$ from a multivariate Normal distribution given the newly sampled $\boldsymbol{\omega}_k$. The derivation of the above equations is given in the next section.

For μ_k, we first sample from the posterior of $\pi_k = \mu_k\left(\phi_k^{-1} + \mu_k\right)^{-1}$, which is Beta:

$$\mathrm{p}(\pi_k|-) = \mathrm{Be}\left(\frac{1}{2} + N\phi_k^{-1}, \frac{1}{2} + \sum_i \tilde{z}_{ik}\right)$$

where $\tilde{z}_{ik} = z_{ik}e^{-\mathbf{X}\boldsymbol{\beta}_k}$. Given π_k, a new value of μ_k is easily derived as: $\mu_k = \pi_k(1-\pi_k)^{-1}\phi_k^{-1}$. A more detailed explanation of the above derivation is given in the next section. Finally, the posterior of ϕ_k is not one of the common distributions and we must again employ a Metropolis step at each iteration of the Gibbs sampler. Proposals ϕ_k^* are sampled from the log-Normal prior of ϕ_k using the most recent values of μ_ϕ and τ_ϕ, and accepted with probability:

$$P_{accept} = \min\left(1, \frac{\prod_i f_i\left(\phi_k^*\right)}{\prod_i f_i\left(\phi_k\right)}\right)$$

where $f_i(\phi_k) = \Gamma(z_{ik} + \alpha_k)\Gamma(\alpha_k)^{-1}(1-\pi_{ik})^{\alpha_k}\pi_{ik}^{z_{ik}}$, with $\alpha_k = \phi_k^{-1}$, $\pi_{ik} = m_{ik}\left(\phi_k^{-1} + m_{ik}\right)^{-1}$ and $m_{ik} = \mu_k e^{\mathbf{X}\boldsymbol{\beta}_k}$. Γ is the gamma function. The hyper-parameters μ_ϕ and τ_ϕ are easily updated by sampling from the

following posteriors:

$$
\begin{aligned}
\mathrm{p}(\mu_\phi|-) &= \mathrm{N}\left(\frac{\sum_k \log\phi_k}{K}, \tau_\phi^{-1}K^{-1}\right)\\
\mathrm{p}(\tau_\phi|-) &= \mathrm{Ga}\left(\frac{K}{2}, \frac{\sum_k(\log\phi_k-\mu_\phi)^2}{2}\right)
\end{aligned}
$$

1.2.5 Further details on the Negative Binomial model

Deriving the conditional posterior $\mathrm{p}(\boldsymbol{\beta}_k|-)$ for the Negative Binomial model requires use of the following identity (Polson *et al.*, 2013):

$$
\begin{aligned}
\frac{(e^\psi)^a}{(1+e^\psi)^b} &= 2^{-b}e^{\kappa\psi}\int_0^\infty e^{-\frac{1}{2}\omega\psi^2}\mathrm{p}(\omega)\mathrm{d}\omega\\
&= 2^{-b}e^{\kappa\psi}\mathbb{E}_\omega\left[e^{-\frac{1}{2}\omega\psi^2}\right] \qquad (1)
\end{aligned}
$$

where $\kappa = a - b/2$ and $\omega \sim \mathrm{PG}(b,0)$ is a random sample from a Polya-Gamma distribution. The implied conditional posterior of ω given ψ is $\mathrm{p}(\omega|\psi) = \mathrm{PG}(b,\psi)$. The above suggests a data augmentation strategy, where we sample ω from its posterior given ψ, followed by sampling ψ, given ω, from the conditional posterior $\mathrm{p}(\psi|\omega) \propto e^{\kappa\psi}e^{-\frac{1}{2}\omega\psi^2}\mathrm{p}(\psi)$, where $\mathrm{p}(\psi)$ is the prior of ψ.

In the case of the Negative Binomial model, the likelihood of a single data point can be written as follows:

$$
\begin{aligned}
\mathrm{p}(z_{ik}|\boldsymbol{\beta}_k) &\propto \left(\frac{\alpha_k}{\alpha_k+\mu_k e^{\mathbf{x}_i^T\boldsymbol{\beta}_k}}\right)^{\alpha_k}\left(\frac{\mu_k e^{\mathbf{x}_i^T\boldsymbol{\beta}_k}}{\alpha_k+\mu_k e^{\mathbf{x}_i^T\boldsymbol{\beta}_k}}\right)^{z_{ik}}\\
&\propto \left(\frac{1}{1+\frac{\mu_k}{\alpha_k}e^{\mathbf{x}_i^T\boldsymbol{\beta}_k}}\right)^{\alpha_k}\left(\frac{\frac{\mu_k}{\alpha_k}e^{\mathbf{x}_i^T\boldsymbol{\beta}_k}}{1+\frac{\mu_k}{\alpha_k}e^{\mathbf{x}_i^T\boldsymbol{\beta}_k}}\right)^{z_{ik}}\\
&\propto \frac{(e^{\psi_{ik}})^{z_{ik}}}{(1+e^{\psi_{ik}})^{z_{ik}+\alpha_k}}\\
&\propto \frac{(e^{\psi_{ik}})^{a_{ik}}}{(1+e^{\psi_{ik}})^{b_{ik}}}
\end{aligned}
$$

where $\psi_{ik} = \log\mu_k - \log\alpha_k + \mathbf{x}_i^T\boldsymbol{\beta}_k$, $\alpha_k = \phi_k^{-1}$, $a_{ik} = z_{ik}$ and $b_{ik} = z_{ik} + \alpha_k$. Then, invoking Bayes' rule and identity (1), the posterior of $\boldsymbol{\beta}_k$ can be written as:

$$
\mathrm{p}(\boldsymbol{\beta}_k|-) \propto \prod_i e^{\kappa_{ik}\psi_{ik}}e^{-\frac{1}{2}\omega_{ik}\psi_{ik}^2}e^{-\frac{1}{2}\boldsymbol{\beta}_k^T\boldsymbol{\Theta}_k\boldsymbol{\beta}_k}
$$

where $\boldsymbol{\Theta}_k = \mathrm{diag}(\tau_k\zeta_{1k}\eta_1,\ldots,\tau_k\zeta_{jk}\eta_j,\ldots,\tau_k\zeta_{jk}\eta_j)$. After some algebra, it follows that:

$$
\mathrm{p}(\boldsymbol{\beta}_k|-) \propto e^{-\frac{1}{2}(\boldsymbol{\beta}_k-\mathbf{m}_k)^T\mathbf{A}_k^{-1}(\boldsymbol{\beta}_k-\mathbf{m}_k)}
$$

which is the kernel of a multivariate Normal distribution with precision matrix $\mathbf{A}_k = \mathbf{X}^T\boldsymbol{\Omega}_k\mathbf{X} + \boldsymbol{\Theta}_k$ and mean vector $\mathbf{m}_k = \mathbf{A}_k^{-1}\mathbf{X}^T\mathbf{y}_k$, where $\boldsymbol{\Omega}_k = \mathrm{diag}(\omega_{1k},\ldots,\omega_{ik},\ldots,\omega_{Nk})$ and $\mathbf{y}_k = \frac{1}{2}\left(\mathbf{z}_k - \phi_k^{-1} - 2\boldsymbol{\omega}_k(\log\mu_k + \log\phi_k)\right)$. The variables ω_{ik} are random samples from a Polya-Gamma distribution $\mathrm{PG}(b_{ik}, c_{ik})$ with parameters $b_{ik} = z_{ik} + \phi_k^{-1}$ and $c_{ik} = \log\mu_k + \log\phi_k + \mathbf{x}_i^T\boldsymbol{\beta}_k$. We sample from the Polya-Gamma distribution as in (Zhou *et al.*, 2012).

Regarding the conditional $\mathrm{p}(\boldsymbol{\mu}_k|-)$, we first compute the posterior of $\pi_k = \mu_k(\alpha_k+\mu_k)^{-1}$ (where $\alpha_k = \phi_k^{-1}$) as follows:

$$
\begin{aligned}
\mathrm{p}(\pi_k|-) &\propto \Pi_i(1-\pi_k)^{\alpha_k}\Pi_i\pi_k^{\tilde{z}_{ik}}\pi_k^{\frac{1}{2}-1}(1-\pi_k)^{\frac{1}{2}-1}\\
&\propto \pi_k^{\sum_i\tilde{z}_{ik}+\frac{1}{2}-1}(1-\pi_k)^{N\alpha_k+\frac{1}{2}-1}\\
&= \mathrm{Be}\left(\sum_i\tilde{z}_{ik}+\frac{1}{2}, N\alpha_k+\frac{1}{2}\right)
\end{aligned}
$$

where $\tilde{z}_{ik} = z_{ik}e^{-\mathbf{X}\boldsymbol{\beta}_k}$. At each iteration, we first sample π_k from the above Beta distribution, and then we calculate $\mu_k = \pi_k(1-\pi_k)^{-1}\phi_k^{-1}$ given the newly sampled π_k.

1.3 Computational considerations

Inference in the above models requires calculating at each iteration the Cholesky decomposition of $\mathbf{A}_k$ for all k, and solving three triangular linear systems involving the Cholesky factor $\mathbf{L}_k$ (a lower triangular matrix) of $\mathbf{A}_k$. Cholesky decomposition has complexity $\mathcal{O}\left(M^3\right)$, while solving each of the triangular systems has complexity $\mathcal{O}\left(M^2\right)$, where M is the number of rows/columns of $\mathbf{A}_k$ (and the number of genetic markers we examine). In order to reduce the computational cost of these calculations, at each iteration we ignore the rows (genetic markers) of $\mathbf{B}_k$ with all elements below a threshold $\beta_{thr} = 10^{-6}$. This effectively reduces the dimensionality of the problem and dramatically increases the speed of the Gibbs sampler. In addition, the above computations are performed in parallel for different k, which further reduces the execution time of the sampler.

1.4 Normalization

When testing *vst* and *voom*, the RNA-seq data (either simulated or natural) are normalized using the default method in each package. For the remaining models, we use the *relative log expression* (RLE) method, which is the default in *DESeq2*.

It should be emphasised that, for eQTL mapping (as for differential gene expression), explicitly normalising read counts with respect to gene length is not necessary. This is because, in both these problems, we wish to identify relative variations in gene expression across a number of samples in all of which each particular gene or transcript has obviously the same constant length. Thus, correcting for gene length is not required. Although logically more reads would map on longer genes for a particular level of expression, the only expected "side-effect" of this would be an increased power to detect variations in the expression of longer genes, due to the presence of a stronger and easier to detect signal (i.e. number of reads).

However, gene length may be important in other applications, which focus on comparison of gene expression within the same sample. In this case, correcting for gene length may be necessary to avoid bias. This can be done using appropriate normalisation methods such as *reads per kilobase of transcript and per million of mapped reads* (RPKM) or *fragments per kilobase of transcript and per million of mapped reads* (FPKM). Laplace smoothing can be subsequently applied on the corrected counts. More generally, any normalisation method that results in a matrix of corrected counts can precede transformation using Laplace smoothing.

1.5 Data simulation

For simulating genotypes, we use data from the *1000 Genomes* project (1000 Genomes Project Consortium *et al.*, 2015). Specifically, we extracted the genotypes for all bi-allelic single nucleotide variants (SNVs) with minor allele frequency (MAF) larger than 5% in the region chr7:100000–200000. This left us with genotypes for 435 SNVs in 2503 samples with known empirical MAFs (Supplementary Figure **7**a). In order to generate a matrix of genotypes $\mathbf{X}$, we select randomly without replacement M SNVs from this dataset and, for each SNV, we use its empirical MAF in a Binomial distribution to generate genotypes for N subjects. For large N, the MAF for each of the M variants matches the empirical MAF of the corresponding variant in the *1000 Genomes* project. For a test case where the genotype data from the *1000 Genomes* project is used as is, see Supplementary Figure **11**.

Ideally, simulation of read counts should not rely on any distributional assumptions. Although there has been some progress in non-parametric simulation of read counts in the context of differential expression studies (e.g. Benidt and Nettleton (2015)), it is still not obvious how to simulate transcript/variant associations (as required in this study) without resorting to some form of parametric model. Given this limitation, we have adopted a Negative Binomial model, which is widely accepted as *the standard approach* for simulating read counts. Towards this aim, we use RNA-seq data from 60 *HapMap* individuals of European descent (Montgomery *et al.*, 2010; Frazee *et al.*, 2011), which includes 11353 genes with non-zero read counts in at least one sample. We fitted a Negative Binomial model to normalized counts of individual genes, thus estimating mean and dispersion parameters for each of them (Supplementary Figure **7**b). In order to simulate a read counts matrix $\mathbf{Z}$, we select randomly without replacement mean and dispersion estimates (μ_k and ϕ_k, respectively) for K genes. Furthermore, we simulate a sparse matrix of regression coefficients $\mathbf{B}$ with non-zero elements $\beta_{jk} \sim \frac{1}{2}(1+e_{jk}) - \frac{1}{2}(1+e_{jk})$, where e_{jk} is a random sample from the Exponential distribution with rate $\lambda = 1$ (Supplementary Figure **7**c). Given the genotypes $\mathbf{X}$, the non-zero elements in the kth column of this matrix are normalized by first dividing with $\max\left(\mid \mathbf{X}\boldsymbol{\beta}_k \mid\right)$ and then multiplying with $\log s$, where $s > 1$ is the maximum fold change in the mean expression level of transcript k across all subjects. This normalization ensures that the fold change for each genes in all subjects is no larger that s or smaller than $1/s$ (Supplementary Figure **7**d). In this paper, we assume that s takes the values 2, 4 or 8 corresponding to *small*, *medium* and *large* effect sizes, respectively.

Finally, given the genotypes $\mathbf{X}$, coefficients $\mathbf{B}$ and $\{\mu_k, \phi_k\}$ estimates for K genes, we can simulate an $N \times K$ matrix of read counts with elements z_{ik} drawn from a Negative Binomial distribution with mean $\mu_k e^{\mathbf{x}_i^T \boldsymbol{\beta}_k}$ and

dispersion ϕ_k (Supplementary Figures **7**e,f).

Further details on the capacity of the Negative Binomial distribution to faithfully model the empirical RNA-seq data are given in Supplementary Figure **8**.

1.6 Performance metrics

The *root mean square error* (RMSE) in Figure 2 is defined as:

$$\text{RMSE} = \sqrt{\frac{\sum_{jk}\left(\beta^{*}_{jk} - \hat{\beta}^{*}_{jk}\right)^2}{\sum_{jk} 1}}$$

where j and k are restricted to the true positive elements of the estimated coefficients matrix $\hat{\mathbf{B}}$, only. The normalized elements β^{*}_{jk} and $\hat{\beta}^{*}_{jk}$ are derived from the elements of the true (β_{jk}) and estimated ($\hat{\beta}_{jk}$) matrices by dividing each with $\sum_{jk} \mid \beta_{jk} \mid$ and $\sum_{jk} \mid \hat{\beta}_{jk} \mid$, respectively. This normalization removes any possible scaling that may have been introduced due to the use of different models, thus making comparisons between them permissible.

For calculating the *concordance correlation coefficient* (CCC) in Figure 3, we treat the expression matrix $\mathbf{Z}$ as an $N \times K$ vector. In the case of Poisson, Binomial and Negative Binomial models, $\mathbf{Z}$ is first log-transformed (after adding 1 to all entries in order to avoid infinities). In all other cases, we use the transformed expression matrix $\mathbf{Y}$ as input. CCC is then defined as:

$$\text{CCC} = \frac{2S_{Y\hat{Y}}}{S^2_Y + S^2_{\hat{Y}} + \left(\bar{Y} - \bar{\hat{Y}}\right)^2}$$

where Y and $\hat{Y}$ indicate test and predicted expression data, respectively. Mean ($\bar{Y}$, $\bar{\hat{Y}}$), variance (S^2_Y, $S^2_{\hat{Y}}$) and covariance ($S_{Y\hat{Y}}$) values are defined as usual.

All other metrics follow their common definitions, as described below. First, for each simulation, we computed the number of *true positives* (TP), *true negatives* (TN), *false positives* (FP) and *false negatives* (FN) by comparing the estimated marker/transcript pairs with zero/non-zero coefficients against the corresponding simulated ones. This comparison is straightforward after reducing the estimated and "true" (i.e. simulated) sparse coefficient matrices ($\hat{\mathbf{B}}$ and $\mathbf{B}$, respectively) to ones with elements in the set $\{-1, 0, 1\}$, which merely indicate the presence/absence and directionality (up- or down-regulation) of a marker/transcript association. Given estimated numbers for TP, TN, FP and FN, we can calculate *Matthews correlation coefficient* (MCC), *true positive rate* (TPR), *true negative rate* (TNR), *positive predictive value* (PPV), *negative predictive value* (NPV) and *accuracy* (ACC) using the following formulas:

$$\begin{aligned}
\text{MCC} &= \frac{TP \times TN - FP \times FN}{\sqrt{(TP+FP)(TP+FN)(TN+FP)(TN+FN)}} \\
\text{TPR} &= \frac{TP}{TP+FN} \\
\text{TNR} &= \frac{TN}{TN+FP} \\
\text{PPV} &= \frac{TP}{TP+FP} \\
\text{NPV} &= \frac{TN}{TN+FN} \\
\text{ACC} &= \frac{TP+TN}{TP+TN+FP+FN}
\end{aligned}$$

2 Supplementary results

2.1 Various metrics

We further test model performance using various additional metrics (Supplementary Figure **1**). These are the *true positive rate* (TPR), *true negative rate* (TNR), *positive predictive value* (PPV), *negative predictive value* (NPV) and *accuracy* (ACC), which is equal to the sum of true positives and true negatives divided by the sum of all positives and negatives. Overall the count-based models have relatively low TPR and PPV at low

to medium samples sizes, but very high TNR and NPV at all sample sizes, which results in a significantly higher accuracy, in relation to Normal models. Among the latter, *arcsin* demonstrates consistently the best performance, particularly at small to medium sample sizes.

2.2 Strength and distribution of gene/variant associations

We examine how model performance is affected by the strength of gene/variant associations (i.e. the effect size), their number and their distribution at small ($N = 250$) and large ($N = 2000$) sample sizes. We test three different effect sizes: *small*, *medium* and *large*, which are defined according to the fold change they induce in gene expression (see precise mathematical definition in *Supplementary Methods*). Regarding the number of associations, we test data with *1*, *2*, *4*, *8*, *16*, *32* and *64* associations and we made sure that both hotspots (i.e. single genetic markers influencing multiple genes) and polygenic effects (i.e. multiple genetic markers affecting the same gene) were included (Supplementary Figure **9**). As illustrated in Supplementary Figure **2**, increasing the effect size has a positive effect on the performance of all models. At small samples and at all effect sizes, the *arcsin* model shows the best performance. At large samples, the Negative Binomial and Poisson models take the lead (along with the *arcsin* model) at small and medium effect sizes. At large effect sizes, the Poisson and *arcsin* models are pulled towards the mean, leaving the Negative Binomial as the model with the best performance. A similar pattern is observed when we examine the number and distribution of gene/variant associations (Supplementary Figure **3**), with the Negative Binomial, Poisson or *arcsin* as the best performing model in most cases.

2.3 The effect of noise

We examine the effect of contaminating the transcript expression data with two different types of noise in the form of extreme outliers (Soneson and Delorenzi, 2013). *Type I* noise was generated by considering each element of the expression matrix **Z** independently and multiplying it, with a probability *50%*, with a random number between *5* and *10*. *Type II* noise was generated by selecting, for each gene, one sample at random and multiplying the counts corresponding to that gene with a random number between *5* and *10*. With *Type I* noise, we expect 50% of the counts in each sample to be outliers, while *Type II* noise ensures that the counts for all genes are surely outliers in one of the samples. *Type I* noise represents a rather extreme scenario compared to *Type II*, although both types of noise include equally strong outliers. As illustrated in Supplementary Figure **4**, *Type I* noise has a negative effect on model performance at all samples sizes, while *Type II* noise has a specific negative effect on the Negative Binomial model at small sample sizes only. The *arcsin* model still shows top performance at small samples in both noise scenarios, but the Negative Binomial model has relatively good performance only in the *Type I* noise scenario. The performance of the Poisson model is either below average (noise *Type I*) or average (noise Type II). At large sample sizes, the Negative Binomial, Poisson and *arcsin* models retain their lead under both noise scenarios.

Furthermore, we examine a second kind of noise, which takes the form of random genotyping errors (Supplementary Figure **5**). We simulate these errors by randomly modifying the number of alleles in a subset of the genotypes matrix **X** to one of their two possible alternatives. For example, if element (i, j) in this matrix is 1 (which indicates a heterozygous call), we randomly change this to either 0 (wild-type homozygous) or 2 (mutant homozygous). We apply this process to either 15% or 30% of the elements of matrix **X**. Not surprisingly, increasing the proportion of genotyping errors reduces the performance of all models, particularly at small samples (N=250), where the average MCC value drops by more than half at 30% error rate. At this error rate, *arcsin*, followed by *nbin*, demonstrates the best performance. At large samples (N=2000), reduction in average MCC values at higher amounts of genotypic noise does not seem to modify relative performance, with *nbin* and *pois* being the best-performing models, closely followed by *arcsin*.

2.4 Violation of model assumptions

Over-dispersion, i.e. the situation where the variance of the read counts for each gene is larger than the mean, is one of the signature characteristics of RNA-seq. In this section, we examine the extreme scenario where only half of the genes are over-dispersed, while the other half has variance equal to the mean, as assumed by the standard (i.e. non-over-dispersed) Poisson model. At small samples (Supplementary Figure **6**), the lack of over-dispersion in a significant proportion of genes severely hits the performance of the Negative Binomial model, but boosts the performance of the Poisson and Normal models smoothing out the difference between them. At large samples, estimating the Negative Binomial model did not finish after 30 hours of computation due to the introduction of many false positives and it was excluded from the analysis. In this scenario, the Poisson model becomes the top-performer followed by the Normal model with either *log*- or *vst*-transformed data. This is the only case we observed, where *arcsin* has smaller performance than other Normal models.

The fact that the Normal model appears robust in violations of basic distributional assumptions is expected, because its application requires transforming the data first (regardless of the form of its original distribution) in order to make it more normal-like. This is an additional point in favour of a transformation-based statistical approach.

References

1000 Genomes Project Consortium, Auton, A., Brooks, L. D., Durbin, R. M., Garrison, E. P., Kang, H. M., Korbel, J. O., Marchini, J. L., McCarthy, S., McVean, G. A., and Abecasis, G. R. (2015). A global reference for human genetic variation. *Nature*, **526**(7571), 68–74.

Benidt, S. and Nettleton, D. (2015). Simseq: a nonparametric approach to simulation of rna-sequence datasets. *Bioinformatics*, **31**(13), 2131–40.

Frazee, A. C., Langmead, B., and Leek, J. T. (2011). Recount: a multi-experiment resource of analysis-ready rna-seq gene count datasets. *BMC Bioinformatics*, **12**, 449.

Montgomery, S. B., Sammeth, M., Gutierrez-Arcelus, M., Lach, R. P., Ingle, C., Nisbett, J., Guigo, R., and Dermitzakis, E. T. (2010). Transcriptome genetics using second generation sequencing in a caucasian population. *Nature*, **464**(7289), 773–U151.

Polson, N. G., Scott, J. G., and Windle, J. (2013). Bayesian inference for logistic models using polya-gamma latent variables. *J. Am. Stat. Assoc.*, **108**(504), 1339–1349.

Soneson, C. and Delorenzi, M. (2013). A comparison of methods for differential expression analysis of rna-seq data. *BMC Bioinformatics*, **14**, 91.

Zhou, M., Li, L., Dunson, D., and Carin, L. (2012). Lognormal and gamma mixed negative binomial regression. *Proc Int Conf Mach Learn*, **2012**, 1343–1350.

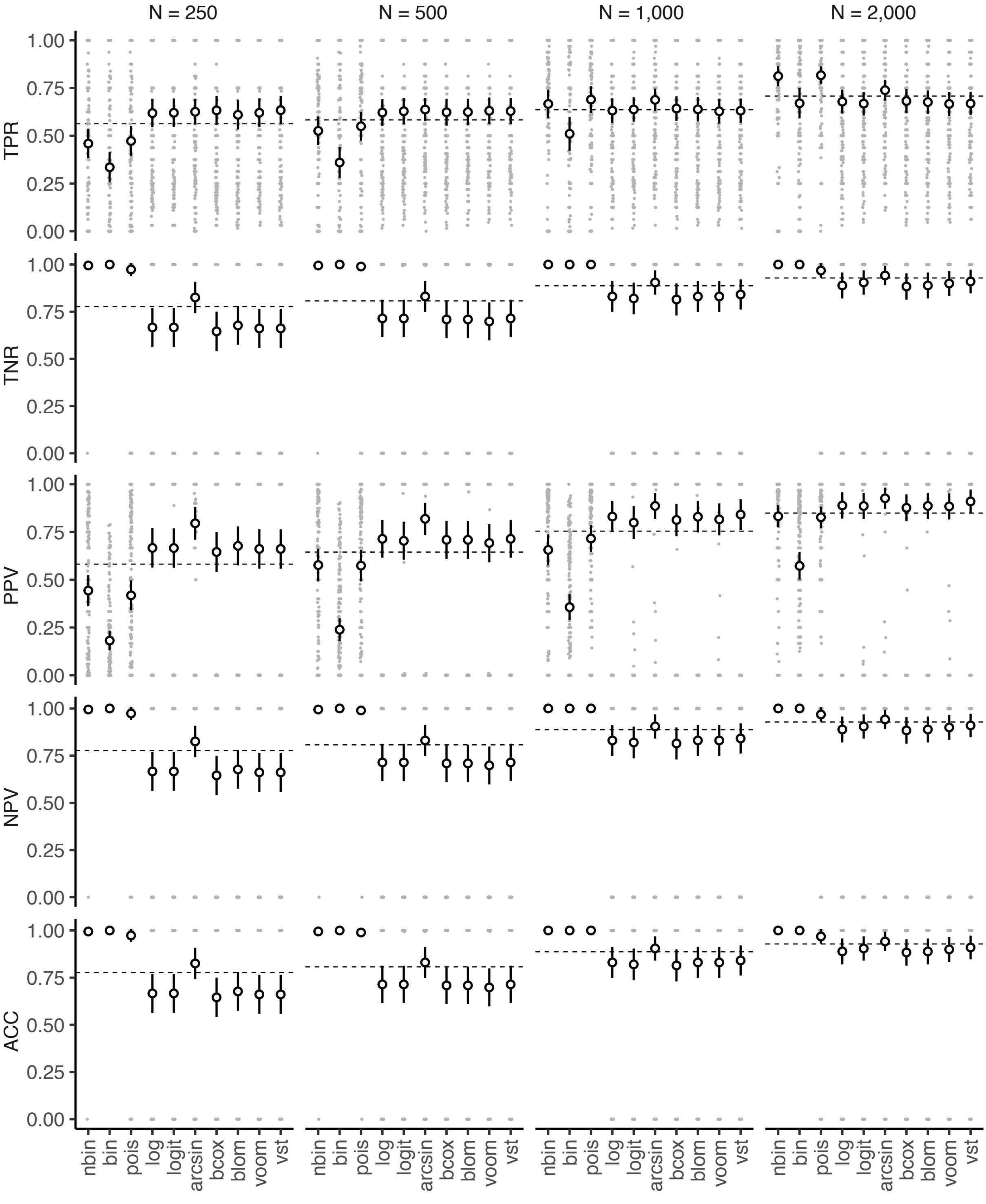

Suppl. Fig. 1: **Model performance assessed by various metrics.** We examine small ($N = 250$), medium ($N = 500, 1000$) and large ($N = 2000$) samples. **TPR:** *true positive rate* or *sensitivity*; **TNR:** *true negative rate* or *specificity*; **PPV:** *positive predictive value* or *precision* (this is equal to 1 minus the *false discovery rate*); **NPV:** *negative predictive value*; **ACC:** *accuracy.*

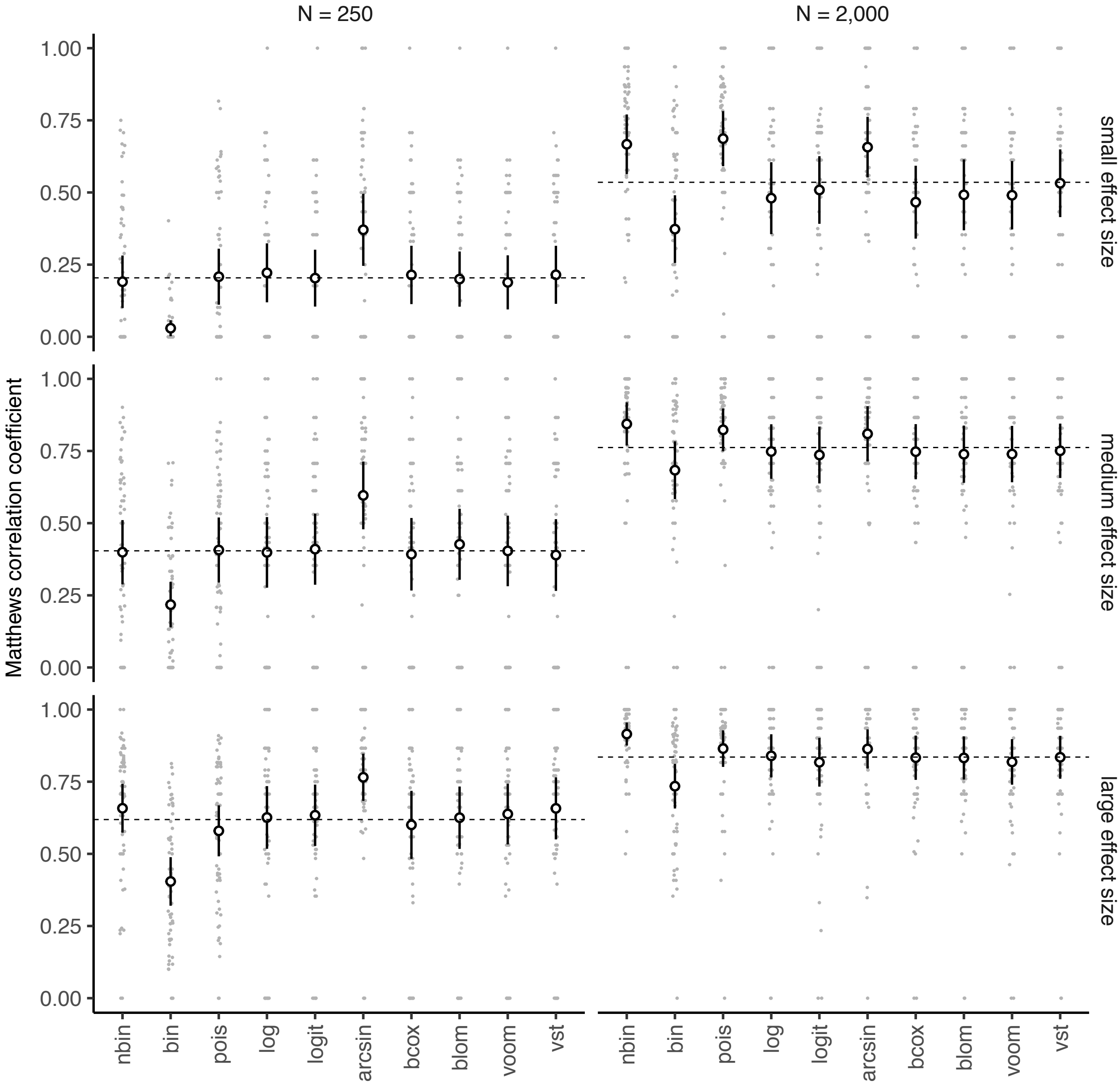

Suppl. Fig. 2: **The effect of gene/variant association strength on model performance.** All models are benchmarked on data with *small*, *medium* and *large* association strengths at both small ($N = 250$) and large ($N = 2000$) sample sizes.

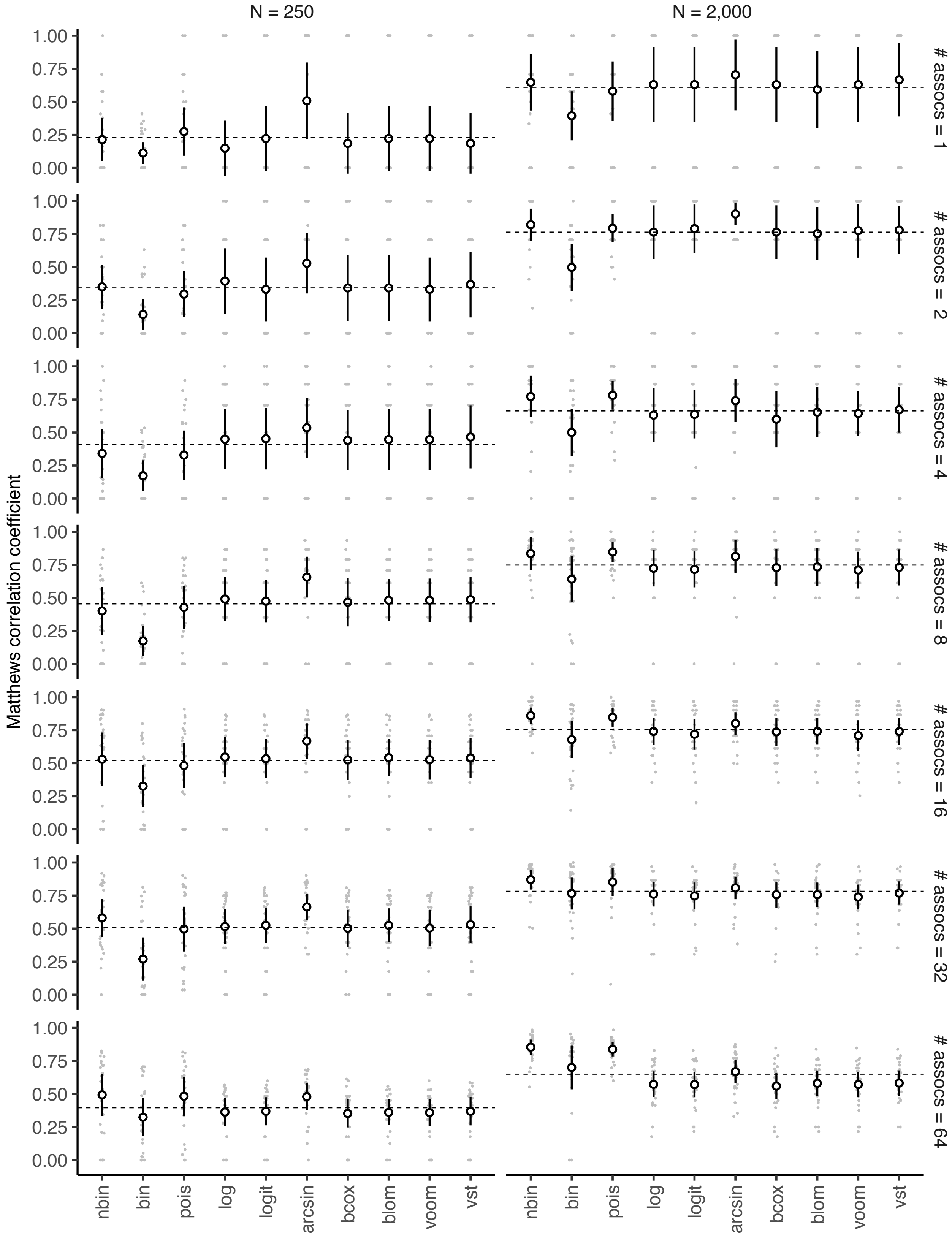

Suppl. Fig. 3: **The effect of number and distribution of gene/variant associations on model performance.** We tested data with *1*, *2*, *4*, *8*, *16*, *32*, and *64* associations at both small ($N = 250$) and large ($N = 2000$) samples. See Supplementary Figure ***9*** for more details on the distribution of associations.

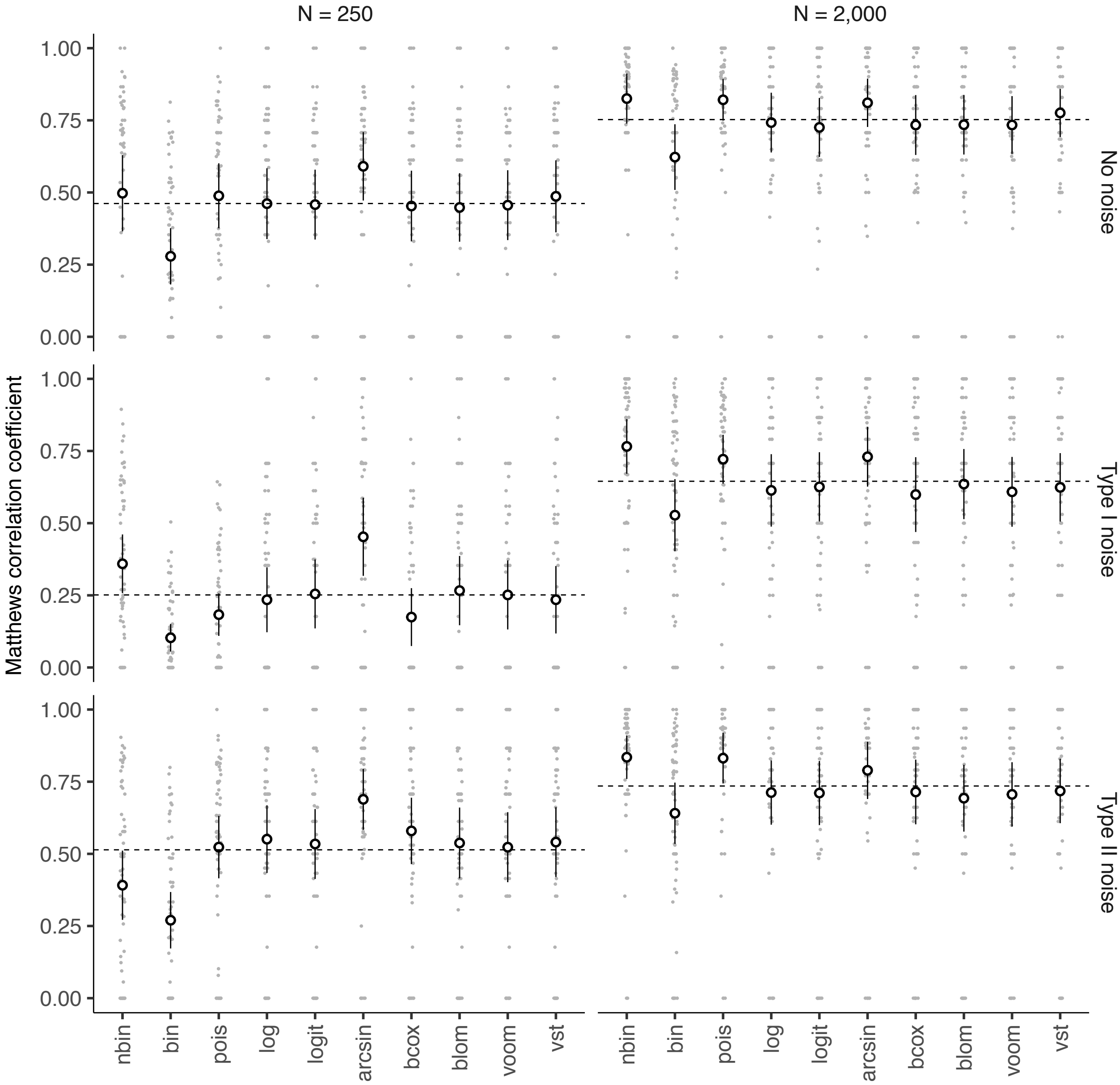

Suppl. Fig. 4: **The effect of transcript expression noise on model performance.** We consider two types of noise, *Type I* and *Type II*, at both small ($N = 250$) and large ($N = 2000$) sample sizes.

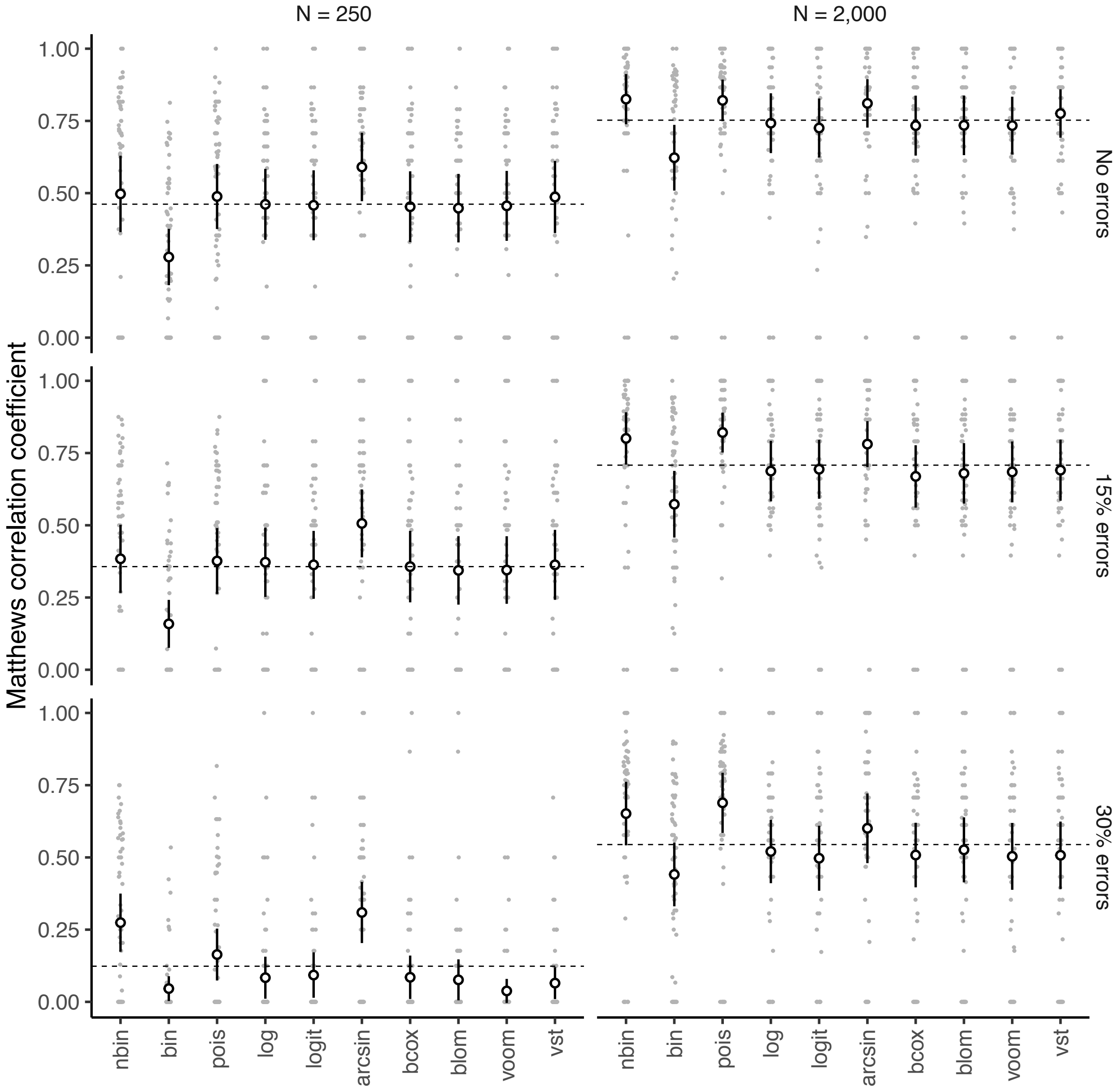

Suppl. Fig. 5: **The effect of genotyping errors on model performance.** A proportion of genotypes (either 15% or 30%) is simulated to be erroneous. We consider this scenario at both small ($N = 250$) and large ($N = 2000$) samples.

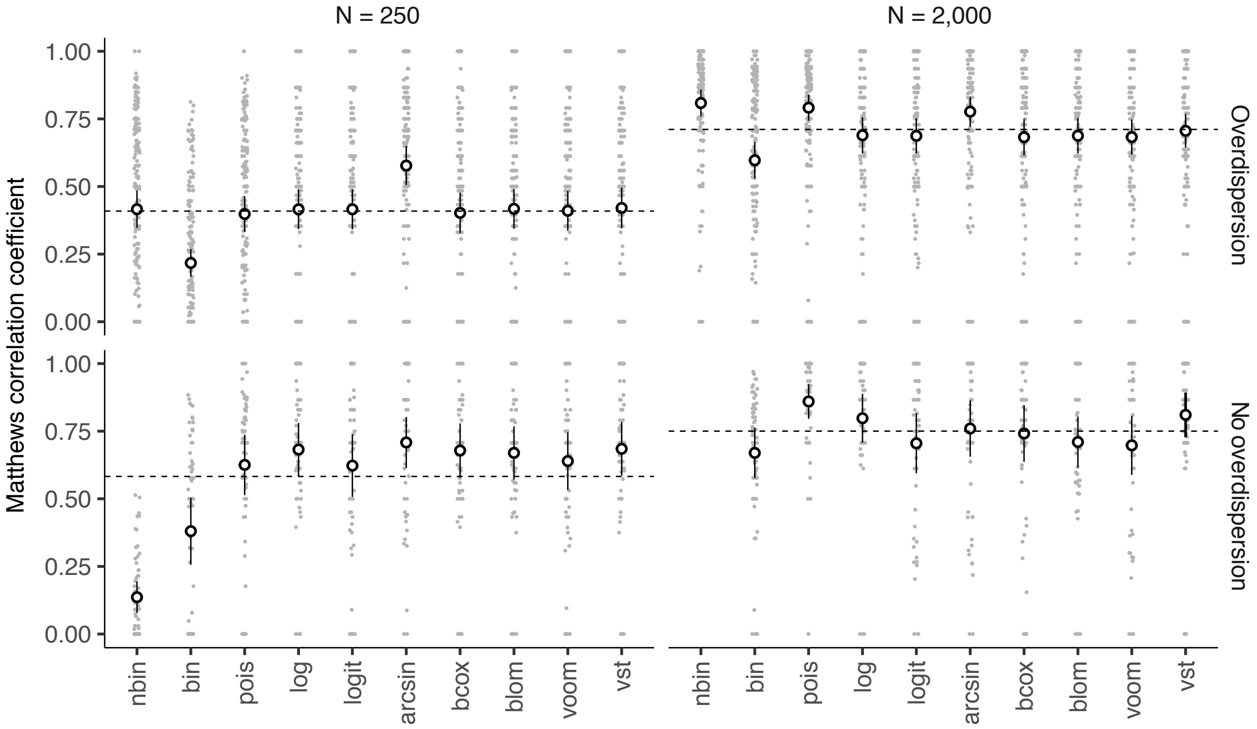

Suppl. Fig. 6: **The effect of over-dispersion on model performance.** We examine the impact on model performance at both small (*N=250*) and large (*N=2000*) samples, when 50% of the genes do not demonstrate over-dispersion, but rather their variance is equal to the mean, as in the standard Poisson model.

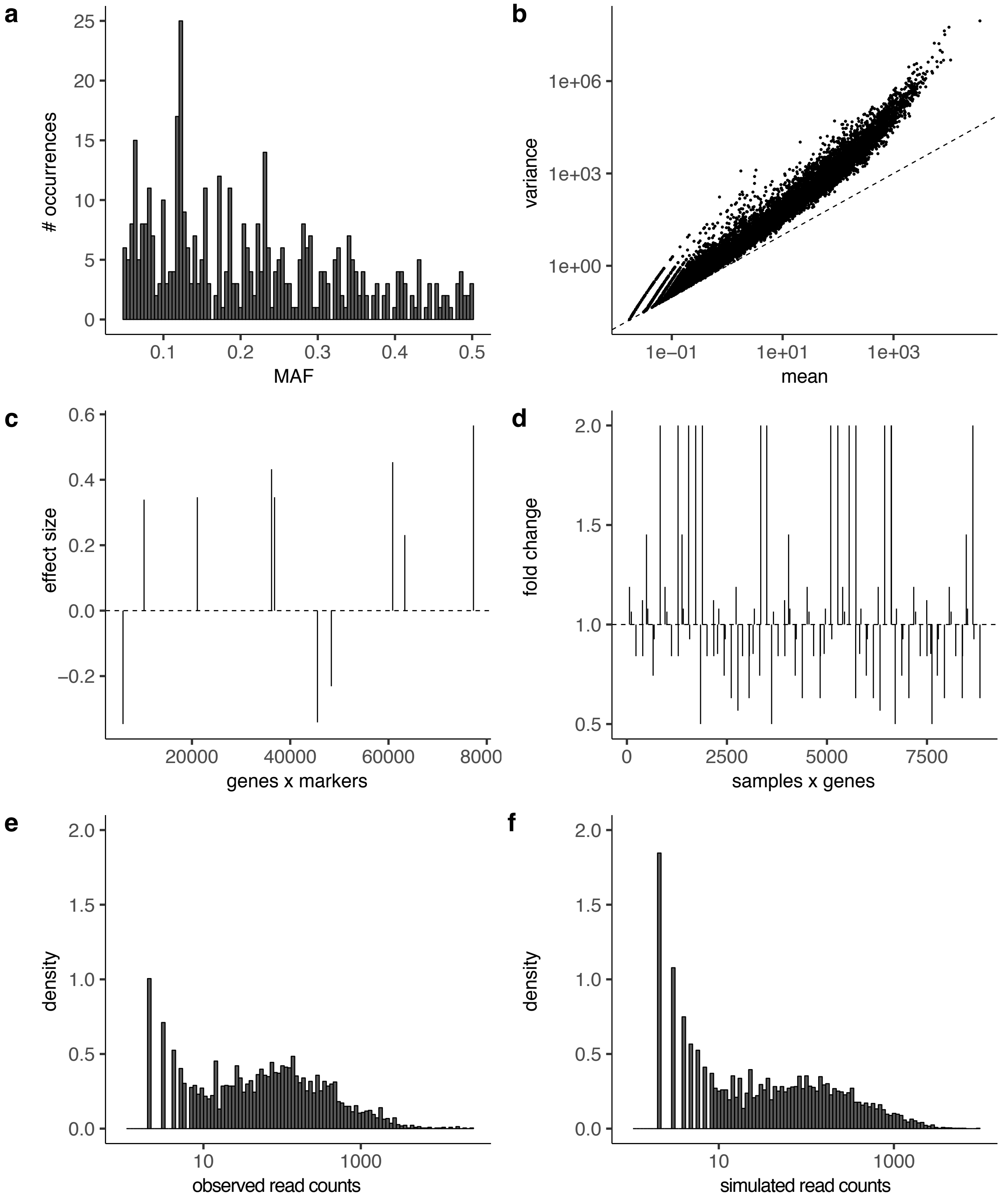

Suppl. Fig. 7: **Data simulation using natural data as template (I).** (a) Distribution of the empirical MAFs for 435 SNVs lifted from chromosome 7 of the *1000 Genomes* project. (b) Mean versus variance estimated from RNA-seq data from 60 *HapMap* individuals of European descent (the **dashed line** indicates points with variance equal to the mean). (c) A simulated matrix of coefficients $\mathbf{B}$ illustrated as a vector of size $M \cdot K$. The matrix includes 10 (7 positive and 3 negative) gene/variant associations. The remaining elements of the matrix are 0. (d) The corresponding matrix of fold-changes in gene expression in relation to the mean across all subjects. This is calculated as $e^{\mathbf{XB}}$, where $\mathbf{X}$ and $\mathbf{B}$ are matrices of simulated genotypes and association coefficients, respectively. Again, the matrix is represented as a vector of size $N \cdot K$. (e) Overall distribution of read counts in 10 of the *HapMap* subjects across 1000 genes. (f) Overall distribution of simulated read counts across 10 subjects and 1000 genes.

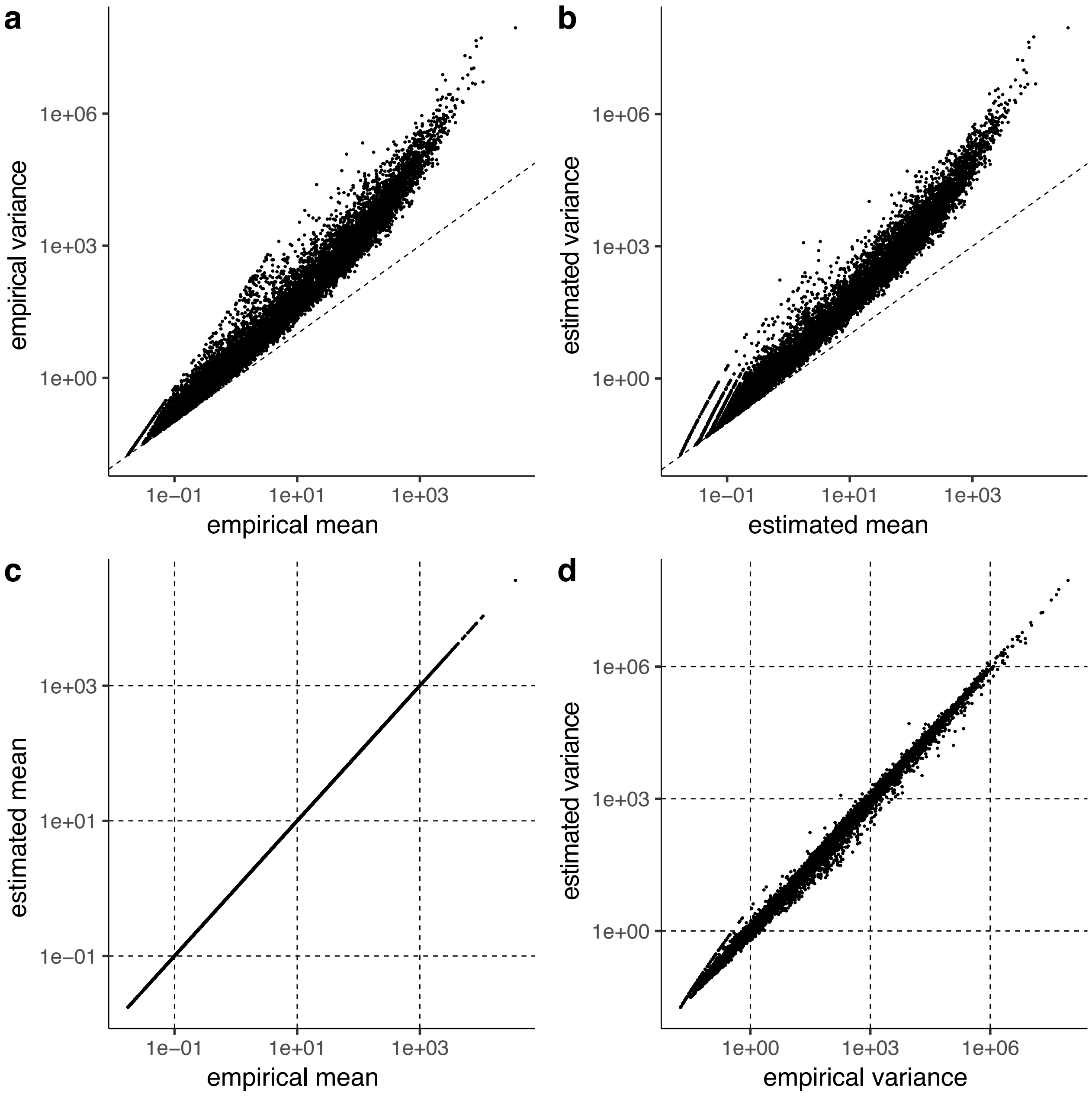

Suppl. Fig. 8: **Data simulation using natural data as template (II).** (a) Empirical mean/variance relation in the RNA-seq data from 60 *HapMap* individuals of European descent. (b) Theoretical mean/variance relation assuming a Negative Binomial model estimated using the data in (a). This is the same as in Supplementary Figure **7**b. (c) Empirical vs theoretical mean transcript expression values in the same data assuming a Negative Binomial model. (d) Empirical vs theoretical transcript expression variance assuming again a Negative Binomial model. Overall, the close correspondence between observed and estimated values indicates that a Negative Binomial model can fit the data with high fidelity.

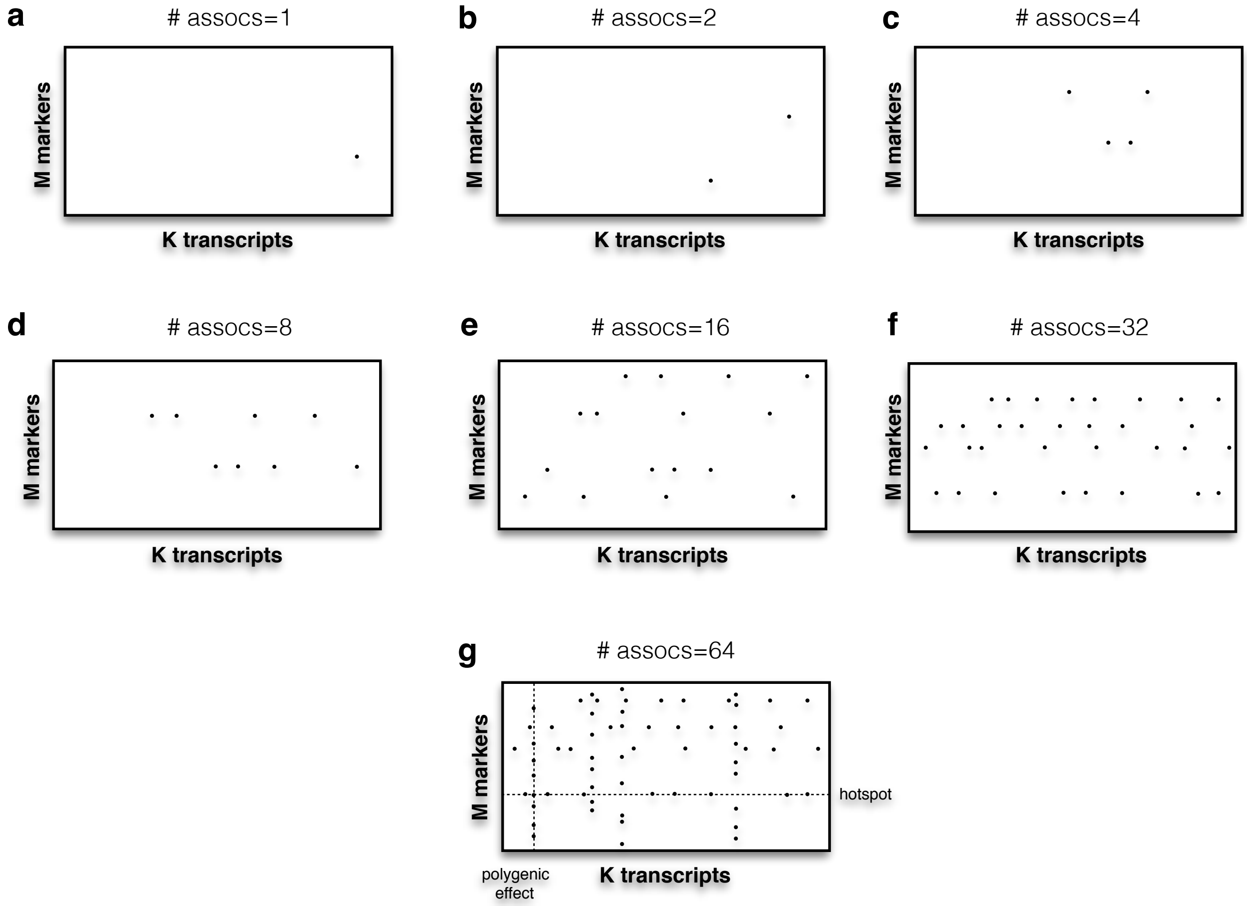

Suppl. Fig. 9: **Distribution of gene/variant associations in simulated data.** We examine data with 1, 2, 4, 8,16, 32 and 64 randomly distributed, non-overlapping associations. (a) A single variant influences the expression of a single gene. (b) Two different variants influence the expression of a single gene each. (c) Two different variants influence the expression of two different genes each. (d) Two different variants influence the expression of four different genes each. (e) Four different variants influence the expression of four different genes each. (f) Four different variants influence the expression of eight different genes each. (g) As in the previous pattern, plus four different genes being influenced by eight different variants each.

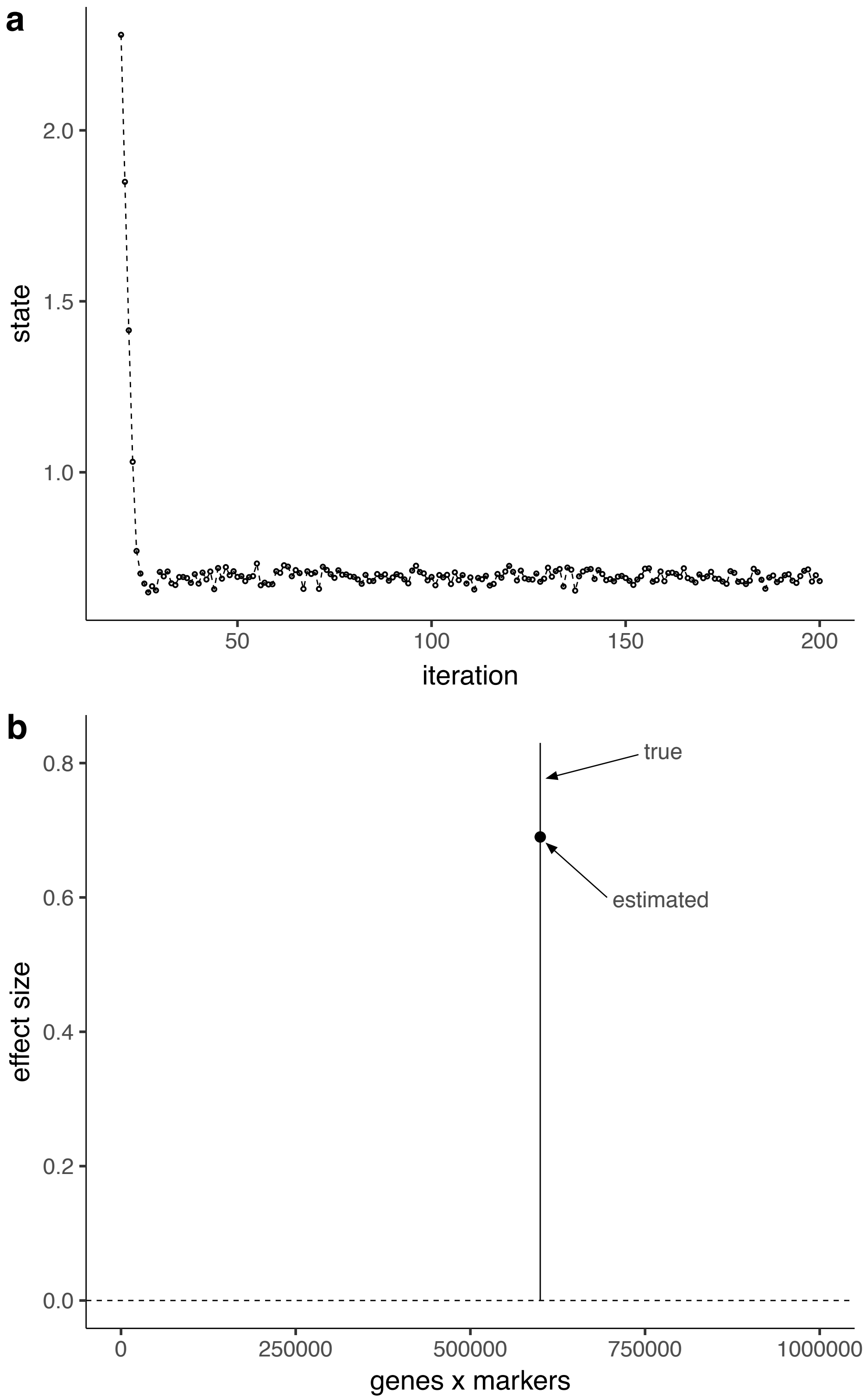

Suppl. Fig. 10: **An example output of *eQTLseq*.** We used *eQTLseq* to process a simulated dataset with 2000 samples, 1000 genes and 1000 markers. The dataset included a single transcript/marker association. The Gibbs sampler was left to run for 2000 iterations. (a) The first 200 iterations of the sampler using the Normal model and a simple logarithmic transformation of the expression data. The sampler reached a steady state quite quickly. It took 21 minutes to complete sampling on an Ubuntu Linux workstation with an Intel Xeon processor utilising four cores and 64GB of memory. (b) The matrix of coefficients $\mathbf{B}$ as a vector with $M \cdot K$ elements. The "true" (solid vertical line) and estimated (black dot) strengths of the simulated marker/transcript association are indicated. The Poisson, Binomial and Negative Binomial models give a similar picture, but they converge slower (>1000 iterations) and sampling is more time-consuming (all three models completed 2000 iterations in 1 hour or more).

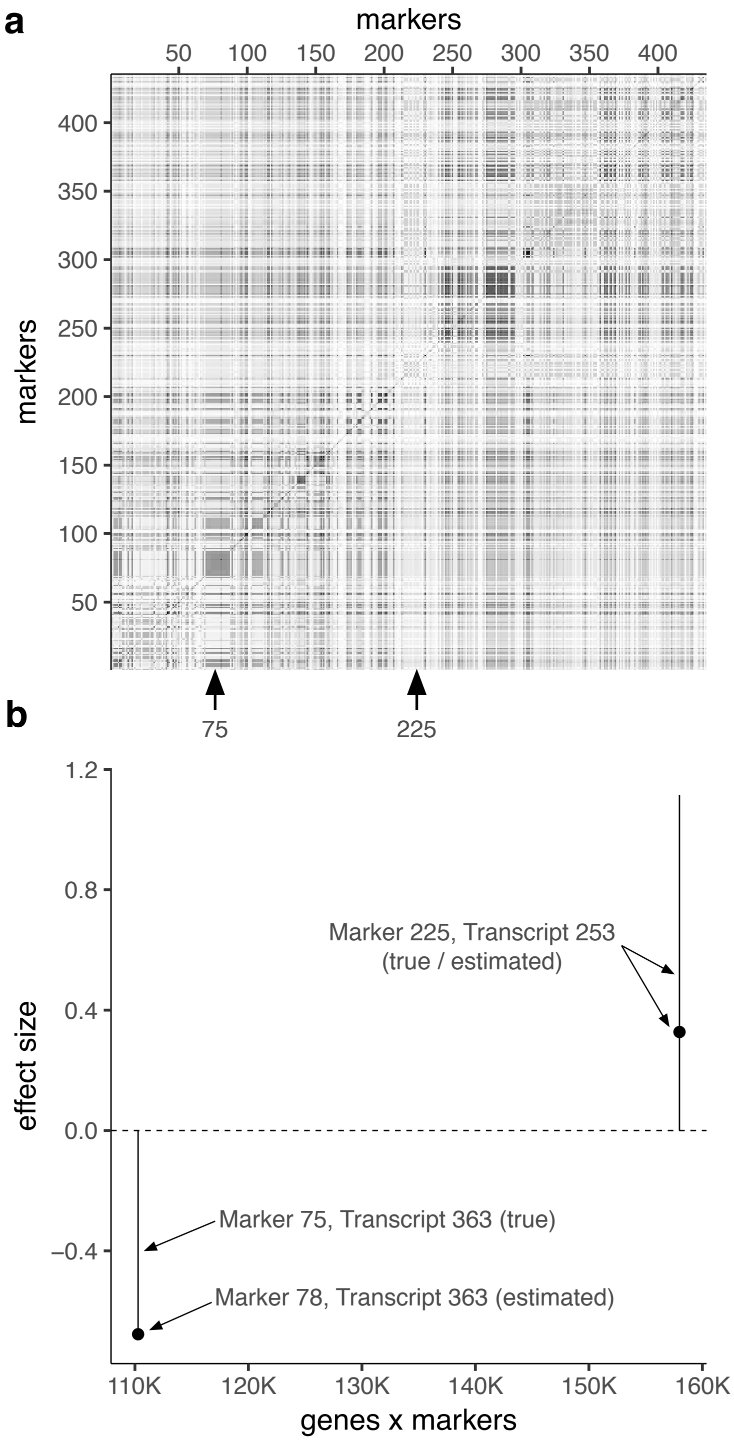

Suppl. Fig. 11: **The effect of correlation between genetic markers.** We examine the scenario where the template matrix of genotypes (see *Data simulation*) is used as is and only the matrix of expression data is simulated. (a) Covariance between all pairs of genetic markers in the data. Marker 75 is embedded in a block of highly correlated markers, while marker 225 is located in a region of relatively uncorrelated markers (see arrows at the bottom). (b) Markers 75 and 225 are associated with transcripts 363 (down-regulation) and 253 (up-regulation), respectively (see vertical solid lines). While eQTLseq correctly identifies marker 225 as an eQTL, it also identifies as such marker 78, instead of the highly correlated marker 75 (see black dots). As with other variable selection methods (e.g. LASSO), when presented with a group of highly correlated variables, eQTLseq selects a single one among them in a possibly non-deterministic manner. In this scenario, we used the Normal model with a logarithmic transformation of the simulated read counts. Although eQTLseq can be used with correlated data, as demonstrated above, its intended use is with pre-filtered genotypic data consisting of mostly uncorrelated markers.
